# The ribosome biogenesis factor yUtp23/hUTP23 coordinates key interactions in the yeast and human pre-40S particle and hUTP23 contains an essential PIN domain

**DOI:** 10.1093/nar/gkw1344

**Published:** 2017-01-13

**Authors:** Graeme R. Wells, Franziska Weichmann, Katherine E. Sloan, David Colvin, Nicholas J. Watkins, Claudia Schneider

**Affiliations:** Institute for Cell and Molecular Biosciences, Newcastle University, Newcastle upon Tyne, NE2 4HH, UK

## Abstract

Two proteins with PIN endonuclease domains, yUtp24(Fcf1)/hUTP24 and yUtp23/hUTP23 are essential for early pre-ribosomal (r)RNA cleavages at sites A0, A1/1 and A2/2a in yeast and humans. The yUtp24/hUTP24 PIN endonuclease is proposed to cleave at sites A1/1 and A2/2a, but the enzyme cleaving at site A0 is not known. Yeast yUtp23 contains a degenerate, non-essential PIN domain and functions together with the snR30 snoRNA, while human hUTP23 is associated with U17, the human snR30 counterpart. Using *in vivo* RNA–protein crosslinking and gel shift experiments, we reveal that yUtp23/hUTP23 makes direct contacts with expansion sequence 6 (ES6) in the 18S rRNA sequence and that yUtp23 interacts with the 3΄ half of the snR30 snoRNA. Protein–protein interaction studies further demonstrated that yeast yUtp23 and human hUTP23 directly interact with the H/ACA snoRNP protein yNhp2/hNHP2, the RNA helicase yRok1/hROK1(DDX52), the ribosome biogenesis factor yRrp7/hRRP7 and yUtp24/hUTP24. yUtp23/hUTP23 could therefore be central to the coordinated integration and release of ES6 binding factors and likely plays a pivotal role in remodeling this pre-rRNA region in both yeast and humans. Finally, studies using RNAi-rescue systems in human cells revealed that intact PIN domain and Zinc finger motifs in human hUTP23 are essential for 18S rRNA maturation.

## INTRODUCTION

**Figure 1.**
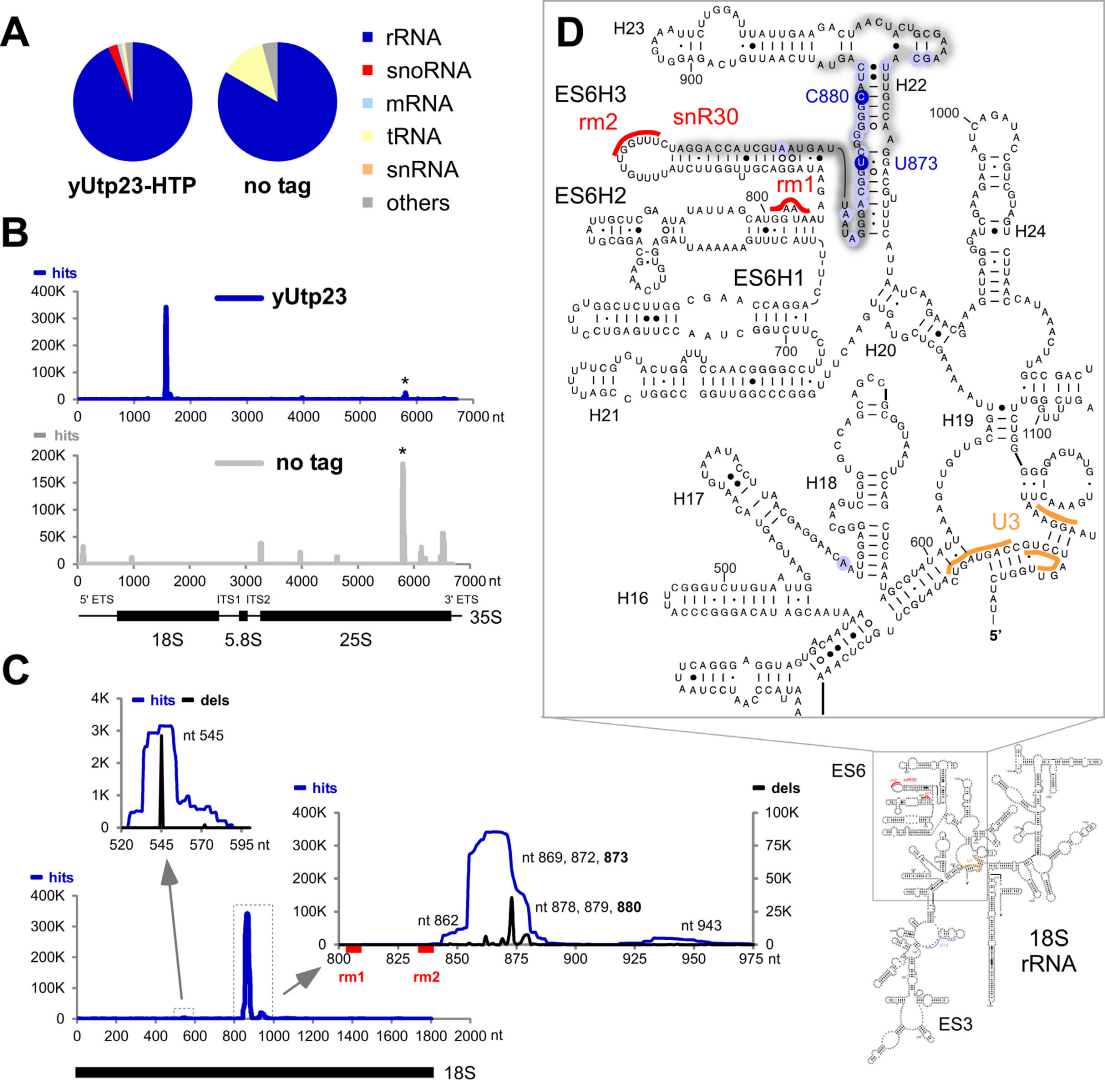
RNA crosslinking sites of yeast yUtp23 on the (pre-) ribosomal RNA. Crosslinked and subsequently trimmed RNAs were purified from yeast strains expressing yUtp23-HTP or a non-tagged control strain and used to generate cDNA libraries. Illumina sequencing data were mapped to the yeast genome using Novoalign. Normalized data for one representative yUtp23-HTP data set and the no tag control are plotted as reads per million mapped sequences (‘hits’). (**A**) Transcriptome-wide binding profiles. A total of 2 277 820 mapped reads were recovered for the yUtp23-HTP data set and 7446 reads for the no tag control. Pie charts illustrate the proportion of all reads mapped to functional RNA classes (indicated on the right). (**B**) yUtp23 crosslinking profile on the primary ribosomal RNA transcript. Sequences were aligned with the rDNA (RDN37-1) encoding the 35S pre-ribosomal RNA. The frequency of recovery in the yUtp23 data set (blue) or the no tag control (gray)is plotted as total reads (hits) for each individual nucleotide. The positions of the mature 18S, 5.8S and 25S rRNAs are indicated by thick lines. A common CRAC contaminant at the 3΄ end of the 25S rRNA is marked by an asterisk (26). ETS: external transcribed spacer; ITS: internal transcribed spacer. (**C**) yUtp23 crosslinks on the 18S rRNA. Hits (blue): total reads; deletions (black; dels): mutations and microdeletions representing precise binding sites. Prominent microdeletion peaks around nt 545 (left insert) and in the 18S ES6 region (right insert) are labeled. The position of the mature 18S rRNA and the snR30 binding sites in the 18S ES6 region (rm1 and rm2) are indicated by a thick black line or red boxes, respectively. (**D**) Predicted secondary structure of the mature 18S rRNA in *S. cerevisiae*. yUtp23 crosslinking sites are marked on the sequence and gray shades indicate peak height. Microdeletion peaks (see panel C) are highlighted by shaded blue circles. Binding sites for the snoRNAs snR30 in the expansion sequence 6 (ES6) region (rm1 and rm2, red) and U3 around the central pseudoknot (orange) are also indicated.

Eukaryotic ribosomal (r)RNAs are processed from an initial 35S (*Saccharomyces cerevisiae*) or 47S (*Homo sapiens*) precursor (pre-rRNA) by a series of endonucleolytic cleavages followed by exonucleolytic trimming, which results in the concomitant removal of external (5΄ ETS, 3΄ ETS) and internal (ITS1, ITS2) transcribed spacer sequences (Figure 1 and Supplementary Figure S1) (1). The early pre-rRNA cleavages, at sites A0, A1 and A2 in yeast and A’, A0, A1/1 and 2a/E in humans, are critical for 18S rRNA maturation and require the small subunit (SSU) processome, a large ribonucleoprotein complex (2). The U3 small nucleolar (sno)RNA, a key component of the SSU processome, base-pairs with the 5΄ ETS and 18S rRNA sequences to guide the formation of the conserved central pseudoknot, which is an essential feature of the 40S ribosomal subunit (2,3).

Many of the factors involved in ribosome biogenesis are essential for pre-rRNA cleavages, which has made the identification of the nuclease activities responsible for these cleavage events difficult. The PIN (PilT N-terminus) endonucleases yUtp24/Fcf1 (hUTP24 in humans) and yNob1 (hNOB1 in humans) are critical for 18S rRNA processing. Early pre-rRNA cleavages at three sites, A0, A1/1 and A2/2a, require the presence of yUtp24/hUTP24 and the PIN endonuclease domain of the protein is essential for cleavages at A1/1 and A2/2a (4–7). The yeast and human proteins also specifically cleave site A2 *in vitro* and yeast yUtp24 was shown to crosslink to both the U3 snoRNA and close to the A1 cleavage site in the pre-rRNA *in vivo* (7). yNob1/hNOB1 functions later in the 18S rRNA processing pathway and catalyzes the removal of the final part of ITS1 from the 3΄ end of the 18S rRNA (site D/3) in the cytoplasm (5,8–11). Only the nucleases that cleave at the A0 and the metazoan-specific A’ sites have yet to be assigned in the 18S rRNA maturation pathway.

In addition to yUtp24/hUTP24, one other PIN domain protein, yUtp23 (hUTP23 in humans), is essential for early cleavages at A0, A1/1 and A2/2a (4,12,13). However, yeast yUtp23 contains only two of the four conserved amino acids in the catalytic site and the two conserved PIN domain residues are not essential for yUtp23 function (4,14). In addition, the protein contains a conserved CCHC Zinc finger. yUtp23 binds nucleotides 745–859 in the 18S rRNA *in vitro* and specifically associates with the snR30 H/ACA snoRNP *in vivo* (14,15). The snR30 snoRNA is essential for the A0, A1 and A2 cleavages and SSU processome assembly (16,17), and is needed for the integration of yUtp23 into the pre-ribosome (15). Conversely, snR30 does not require yUtp23 for pre-ribosome association, but yUtp23 is essential for snR30 release from the complex (15). The internal loop in the 3΄ hairpin of snR30 base-pairs with two elements (rm1 and rm2) in the 18S rRNA expansion sequence 6 (ES6) and is expected to play an important role at this region during pre-SSU maturation (18,19). Interestingly, yUtp23 may be needed to establish base-pairing of snR30 with the 35S pre-rRNA (15). A number of other factors interact with snR30 and/or ES6 including yRok1 (snR30 and ES6 (20)), yRrp7 (ES6 (21)), yUtp24 (ES6 (7)) and yRrp5 (snR30 and ES6 (22)). Release of snR30 from the pre-ribosome requires the RNA helicase yRok1, which directly interacts with yRrp5 (22–24).

Considerably less is known about UTP23 in *Metazoa* but similar to the yeast protein, it is required for pre-rRNA cleavages in the 5΄ ETS and ITS1 (at sites A0, 1 and 2a in humans and mice) (12,13) and is also associated with the human homologue of snR30, U17 (15). However, hUTP23 has three of the four key acidic amino acids of the PIN domain, together with the Zinc finger motif, suggesting that it may be an active nuclease in human cells, but why such activity would be required in humans, but not in yeast, remains unclear.

Here, we present a combination of *in vivo* and *in vitro* approaches to determine the role of yUtp23/hUTP23 in both yeast and human ribosome biogenesis. *In vivo* RNA-protein crosslinking studies (CRAC) generated a transcriptome-wide RNA binding profile for yeast yUtp23, which provides new insights into its relationship with snR30 and the ES6 region of the 18S rRNA. We also performed *in vitro* assays using recombinant proteins to determine RNA and protein interaction partners of yeast yUtp23 and human hUTP23 in the SSU processome. Finally, we established RNAi-rescue systems in HEK293 cells to study the effect of mutant hUTP23 on pre-rRNA cleavage in the human system.

## MATERIALS AND METHODS

### Yeast strains and methods

The *S. cerevisiae* strain expressing genomically encoded, C-terminal HTP-tagged (His_6_-TEV-protA) yUtp23 under the control of its endogenous promoter (Supplementary Table S1) was constructed by standard methods. Cultures were grown at 30°C in medium containing 2% glucose and 0.67% nitrogen base.

### CRAC and data analysis

Actively growing yeast cultures in SD medium (OD_600_ ∼0.5) were UV-irradiated in a 1.2 m metal tube for 100 s at 254 nm to generate RNA–protein crosslinks. The CRAC method (Supplementary Figure S2A) was performed as described in (25,26). Illumina sequencing data were aligned to the yeast genome using Novoalign (http://www.novocraft.com). Sequencing data from this publication were analyzed as previously reported (27) and submitted to the GEO database (http://www.ncbi.nlm.nih.gov/geo/, identifier GSE87238).

### Cloning and mutagenesis

The open reading frames of the protein genes listed in Supplementary Tables S2 and S3 were amplified from yeast genomic DNA or human cDNA adding restriction sites and cloned into pET100 vectors (Invitrogen). The constructs were either used directly or sub-cloned into different protein expression vectors to purify affinity-tagged recombinant proteins from *Escherichia coli* using standard techniques or used for *in vitro* translation with [^35^S] methionine (TNT, Promega).

A human hUTP23 cDNA construct (generated by IDT) containing a C-terminal His_8_-PP (PreScission protease recognition site)-2xHA (hemagglutinin) tag was amplified by PCR and cloned into the pcDNA5/FRT/TO vector (Invitrogen) for protein expression under the control of a tetracycline-inducible promoter. The coding sequence of hUTP23 was altered to render it resistant to the siRNAs used to deplete the endogenous mRNA (Supplementary Table S4). Point mutations were generated by site-directed mutagenesis using overlapping primers (Supplementary Table S3) and confirmed by sequencing.

### *In vitro* RNA binding electromobility shift assay (EMSA)

Recombinant proteins were expressed and purified as described previously (28). RNA substrates were transcribed in the presence of [^32^P]-αUTP, using plasmid constructs or PCR products with T7 promoter sequences as template (Supplementary Tables S2 and S3). RNA binding assays were performed using trace amounts of radiolabeled RNA in 10 mM Tris/HCl pH 7.6, 75 mM NaCl, 2 mM dithiothreitol, 100 ng/μl bovine serum albumin, 0.8 units/μl RNasin, 4.5% glycerol, 0.05% Tween20 and 500 ng/μl *E. coli* tRNA. A total of 10 μl or 15 μl reactions containing 100–5000 nM protein were incubated for 10 min at 30°C, followed by 10 min incubation on ice, and 2 μl or 3 μl native agarose loading dye (30% glycerol and 0.3% Orange G (w/v)) was added. Products were resolved on 4% polyacrylamide/1x TBE (5% glycerol) native gels and visualized using a Typhoon FLA9000 PhosphorImager.

### Protein–protein interaction studies

GST-bait proteins were immobilized on glutathione sepharose pre-blocked with bovine serum albumin and incubated with His-tagged recombinant proteins or [^35^S] *in vitro* translates for 1 h at 4°C in Buffer NB (20 mM Tris/HCl pH 7.6, 150 mM NaCl, 8.7% glycerol and 0.1% Tween20). The beads were washed five times with buffer NB to remove unbound material. Retained proteins were separated by sodium dodecyl sulphate-polyacrylamide gel electrophoresis (SDS-PAGE) and visualized by Coomassie staining, immunoblotting or by using a PhosphorImager, respectively.

### Cell culture and RNAi

hUTP23-His_8_-PP-2xHA-pcDNA5 constructs or the empty pcDNA5 plasmid were transfected into Flp-In T-Rex HEK293 cells as described by the manufacturer (Invitrogen) and stably transfected cells were cultured according to standard protocols. Expression of exogenous proteins was induced by addition of tetracycline (WT: 0.1–1 ng/µl; D31N: 50–100 ng/µl; C103A: 100–200 ng/µl). For RNAi-mediated depletion of endogenous proteins, cells were transfected with siRNA duplexes (Supplementary Table S4) using Lipofectamine RNAiMAX transfection reagent (Invitrogen) and harvested after 72 h.

### RNA analysis

TRI reagent (Sigma-Aldrich) was used to extract RNA from HEK293 cell pellets. For northern blot analysis, 2 μg of total RNA was separated on 1.2% glyoxal–agarose gels, transferred to nylon membrane and hybridized with [^32^P] 5΄ end radiolabeled oligonucleotides (Supplementary Table S3). Results werevisualized with a Typhoon FLA9000 PhosphorImager and quantified using the ImageQuant software (GE Healthcare).

### Immunofluorescence

HEK293 cells expressing HA-tagged hUTP23 proteins were grown on coverslips and induced with tetracycline for 72 h before being fixed with phosphate buffered saline (PBS) containing 4% paraformaldehyde. Immunofluorescence analysis was performed as described in (5). Briefly, cells were permeabilized using 0.2% Triton (v/v) and then incubated with primary and secondary antibodies (Supplementary Table S5) diluted in 10% fetal calf serum, PBS, 0.1% Triton (v/v) and washed with PBS, 0.1% Triton (v/v). In the final step, cells were washed with PBS containing DAPI (4΄,6΄-diamidino-2-phenylindole, 1:10,000) and mounted onto a slide using Mowiol. Images were obtained using a Zeiss Axiovert 200 microscope with Plan-Apochromat x100 1.4NA objective, Axiovision software and an Axiocam monochrome camera and processed in Photoshop (Adobe).

### Immunoprecipitation

Immunoprecipitation experiments were performed with sonicated whole-cell extracts as previously described (29), with the exception that anti-HA antibody-coupled agarose beads were used. Co-precipitated RNA was extracted, separated by denaturing polyacrylamide electrophoresis and analyzed by northern blotting using [^32^P] 5΄ end radiolabeled oligonucleotides (Supplementary Table S3).

## RESULTS

### Yeast yUtp23 crosslinks to the 18S ES6 region and to the snR30 snoRNA

**Figure 2.**
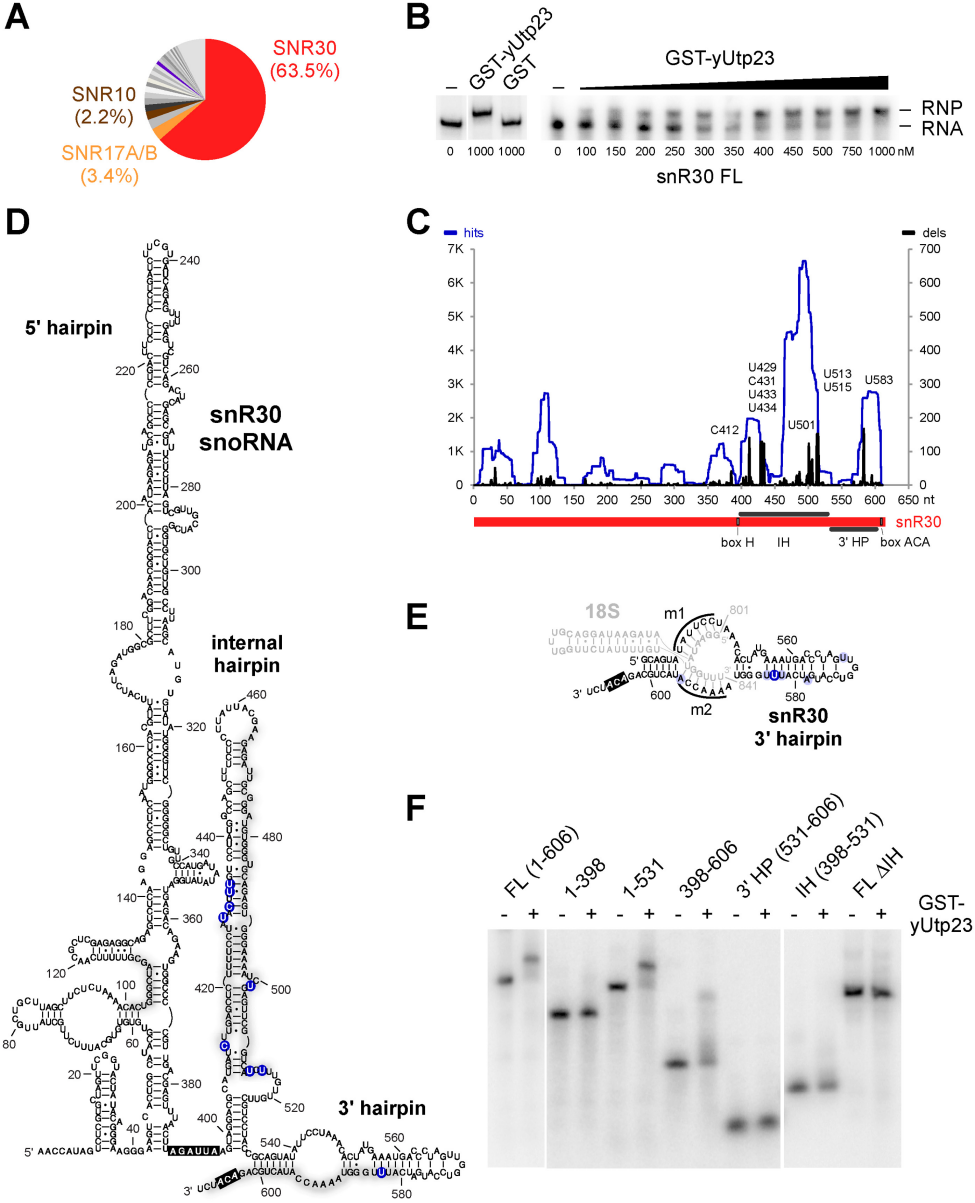
Yeast yUtp23 contacts the internal and 3΄ terminal hairpins of the snR30 snoRNA. (**A**) Pie chart of snoRNA reads from yUtp23 crosslinking data sets. The top 20 hits from two combined yUtp23 data sets (see Supplementary Figure S4B for more detail) are represented as percentages of all reads mapped to snoRNAs. Significant and reproducibly enriched RNAs with essential, non-modifying roles in 18S maturation (snR30, U3 and snR10) are highlighted. (**B**) Electromobility shift assay (EMSA) showing the binding of GST-yUtp23 or GST (1000 nM, left) or GST-yUtp23 (100–1000 nM, right) to *in vitro* transcribed radiolabeled snR30 RNA. Free RNA and RNP complexes were separated on 4% native polyacrylamide gels and visualized using a PhoshorImager. (**C**) yUtp23 crosslinking profile on the snR30 snoRNA. Hits (blue): total reads; deletions (black; dels): mutations and microdeletions representing precise binding sites. Prominent microdeletion peaks in the internal hairpin (IH) and 3΄ terminal hairpin (3΄ HP) are labeled. The positions of the H and ACA boxes within the snR30 RNA are indicated. (**D**) Predicted secondary structure of the snR30 snoRNA in *S. cerevisiae* (adapted from (18)). yUtp23 crosslinking sites are marked on the sequence and grey shades indicate peak height. Predominant microdeletion peaks (see panel C) are highlighted by shaded blue circles. The H and ACA boxes are shown in black. (**E**) A model of the interaction between the 18S ES6 binding sites (m1 and m2) in the 3΄ terminal hairpin (3΄ HP) of snR30 (black) and the 18S ES6 region (nt 801–841, grey). yUtp23 reads (grey) and microdeletion sites in the 3΄ HP (light and dark blue circles) are indicated. Shades of blue represent peak height (see panel C). The ACA box is highlighted in black. (**F**) EMSA showing the binding of GST-yUtp23 (1300 nM) to full-length (FL) snR30 or snR30 RNA fragments. Free RNA and RNP complexes were analyzed as in panel B. IH: internal hairpin; FL ΔIH: full-length snR30 lacking the internal hairpin.

In yeast, yUtp23 is essential for 18S rRNA maturation (4) and required for dissociation of the snR30 small nucleolar RNP from pre-ribosomal particles (15), but its exact role within the SSU processome is unclear. We therefore applied *in vivo* RNA-protein crosslinking (26) to determine yUtp23 RNA binding sites (see Supplementary Figure S2A for overview of the CRAC procedure). We first constructed a yeast strain expressing genomically encoded, C-terminal HTP-tagged (His_6_-TEV-protA) yUtp23 protein under the control of its endogenous promoter. The affinity-tagged yUtp23 protein supported wild-type growth and actively growing yeast cultures were UV-irradiated as described (25). Purification of yUtp23 proteins was verified by western analysis (Supplementary Figure S2B) and co-purified crosslinked RNA fragments were isolated and analyzed as outlined in Supplementary Figure S2A. We reproducibly detected yUtp23 RNA crosslinking sites in four independent CRAC experiments. Results from the two largest data sets (>2 million mapped reads each) are presented in Figures 1 and 2 and Supplementary Figure S4.

Figure 1A shows the transcriptome-wide RNA binding profile of yUtp23 compared to a non-tagged control strain. The majority of reads (>93%) in all four yUtp23 data sets were mapped to rRNA sequences, with lower numbers of hits in snoRNAs (on average ∼2%). Importantly, no snoRNAs hits were detected in the non-tagged control data set. Within the primary 35S pre-rRNA transcript, yUtp23 crosslinks were predominately found in the 18S rRNA sequence (Figure 1B). This crosslinking profile is in agreement with the known function of yUtp23 in 18S rRNA maturation and was not seen with the non-tagged control strain.

The main crosslinking peaks in the 18S rRNA sequence (Figure 1C) are located between 18S nt 844–887 and 18S nt 929–953 of the eukaryotic ES6 or directly downstream of this region (helix 22 and 23, respectively) with some reads also found between 18S nt 534–554 (helix 17 and helix 18). To validate the main crosslinking site in the ES6 region, we performed *in vitro* RNA binding studies with recombinant GST-tagged yUtp23 protein expressed in *E. coli* and a radiolabeled 18S rRNA fragment encompassing the crosslinking sites (18S nt 775–963) (Figure 1D and Supplementary Figures S2C and S2D). Consistent with previously published data (14), the yUtp23 protein (but not the GST-only control) bound to this RNA fragment, albeit with very low efficiency. However, the formation of the RNP complex was specifically decreased by addition of a cold competitor RNA of the same sequence, but not an unrelated RNA fragment of similar size (18S nt 1022–1146). This result indicates specific binding of yUtp23 to this region of the rRNA.

During preparation of the cDNA libraries used for sequencing, microdeletions and/or mutations are often introduced at the site of crosslinking and can therefore be exploited to determine precise protein contact sites (30). Major sites of microdeletion within the main 18S rRNA peaks are highlighted in the inserts shown in Figure 1C. Positioning of the predominant yUtp23 reads and microdeletion sites on the predicted structure of the mature 18S rRNA (Figure 1D) revealed that the main yUtp23 binding sites are in close proximity, but not overlapping with the known snR30 binding sites rm1 (18S nt 801–806) and rm2 (18S nt 836–842) in the ES6 region. Direct yUtp23 protein–RNA contacts within this region were also detected by primer extension analysis on non-digested RNA that was crosslinked to and co-purified with yUtp23 (Supplementary Figure S3A). Notably, similar protein–RNA contacts were also observed between hUTP23 and the human ES6 region when HEK293 cells, expressing an affinity-tagged version of hUTP23, were used for crosslinking and primer extension analysis (Supplementary Figure S3B). This indicates that the interaction between yUtp23/hUTP23 and the 18S ES6 region is evolutionarily conserved.

We next examined the yUtp23 crosslinking sites on snoRNAs (Figure 2 and Supplementary Figure S4). For this, the data for the 20 snoRNAs, to which the maximum number of reads mapped in two independent yUtp23 data sets, were combined and are represented as percentages of reads mapped to all yeast snoRNAs (Figure 2A). Remarkably, 63.5% of snoRNA hits mapped to a single box H/ACA snoRNA, snR30. Significant proportions of reads were also reproducibly mapped to two of the three other snoRNAs involved in 18S rRNA processing (Supplementary Figure S4A), the box C/D snoRNA U3/snR17A/B (3.4%) and the H/ACA snoRNA snR10 (2.2%), whereas reads corresponding to the third, the box C/D snoRNA U14/snR128, were only recovered in one of the analyzed data sets (Supplementary Figure S4B). Hits were also seen for the box C/D snoRNAs snR190 (2.7%) and other modification snoRNAs (0.4–1.8%). The substantial enrichment of snR30 over other snoRNAs suggests that the previously observed relationship between yUtp23 and snR30 involves a direct protein–RNA interaction. Consistent with this, recombinant GST-tagged yUtp23 protein specifically bound to *in vitro* transcribed snR30 RNA, with an estimated dissociation constant (K_D_) value of ∼300 nM (Figure 2B). yUtp23 crosslinking hits and microdeletions could also be mapped to the U3/snR17A/B, snR10 and U14/snR128 snoRNA sequences suggesting direct protein–RNA contacts *in vivo* (Supplementary Figure S4C). However, stable interactions with these RNAs were not detected *in vitro* (data not shown) and might therefore require the presence of the pre-rRNA or other factors within the pre-ribosomal particle.

The positions of yUtp23 crosslinking reads and microdeletion sites on the snR30 sequence (Figure 2, panels C and D) suggest that yUtp23 mainly contacts the 3΄ portion of the snoRNA. Most reads and deletions mapped to the internal hairpin (IH, nt 398–531) and the 3΄ terminal hairpin (3΄ HP, nt 532–601) of snR30, whereas fewer reads were found in the 5΄ hairpin. The high number of yUtp23 crosslinking sites mapping to the internal hairpin was surprising given that this snR30 region was shown to be dispensable for growth *in vivo* (18). Interestingly, the majority of the precise crosslinking sites on the snR30 3΄ HP did not overlap with the snR30 sequences (m1 and m2) that can engage in base-pairing interactions with the 18S rRNA (Figure 2E). Instead, most microdeletions were located within the distal region of the 3΄ HP, which was previously proposed to be a putative snR30-specific snoRNP protein binding site (19).

To investigate the importance of the snR30 IH and 3΄ HP with respect to yUtp23 association, we designed a series of snR30 fragments and tested them for binding to recombinant yUtp23 proteins *in vitro* (Figure 2F). yUtp23 efficiently bound the full-length (FL) snR30 (nt 1–606). In agreement with the CRAC data suggesting significant contacts to the internal hairpin (IH), binding of yUtp23 was severely reduced in the absence of the IH (FL ΔIH). yUtp23 exhibited no binding to the snR30 5΄ hairpin alone (nt 1–398), while binding was restored when the IH (nt 1–531) was included. Moreover, neither the IH (nt 398–531), nor the 3΄ HP (nt 531–606) alone were sufficient for binding, whereas combination of both (nt 398–606) enabled binding, but at a reduced level. The data suggest that the internal hairpin and 3΄ hairpin in snR30 are both needed for yUtp23 binding *in vitro* and that elements in all three hairpins are necessary for maximally efficient binding.

We conclude that yUtp23 primarily binds to the 3΄ portion of the snR30 snoRNA and adjacent to the snR30 base-pairing sites in the mature 18S rRNA sequence. These findings support a direct role for yUtp23 in snR30 function and release.

### Yeast yUtp23 and human hUTP23 directly bind proteins that interact with the 18S rRNA ES6 region and snR30/U17

**Figure 3.**
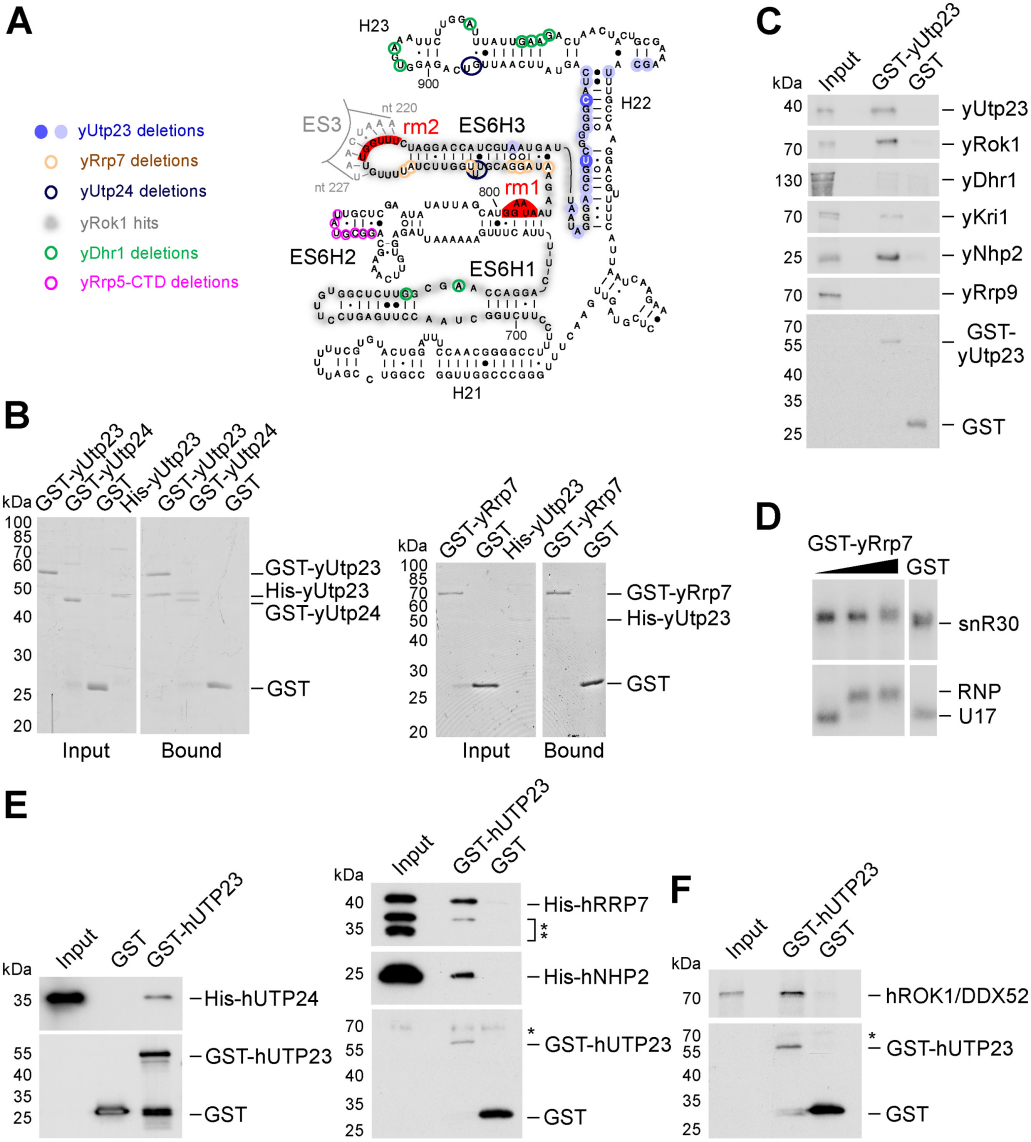
Yeast yUtp23 and human hUTP23 interact with early pre-40S factors contacting the 18S rRNA ES6 region. (**A**) Predicted secondary structure of the mature yeast 18S ES6 region with binding sites of crosslinked ribosome biogenesis factors. Precise crosslinking sites (microdeletions) of yUtp23 (shaded blue circles indicating peak height, see Figure 1C and D) and other published factors (yUtp24 (7), dark blue; yRrp7 (21), brown; yDhr1 (31), green and the yRrp5-CTD (22), pink) are highlighted by circles. yRok1 crosslinking regions (total reads, (20) are shaded in grey. Binding sites for snR30 (rm1 and rm2) are indicated in red. (**B**) Recombinant GST-tagged yeast yUtp23, yUtp24, yRrp7 or free GST were immobilized on glutathione sepharose and incubated with protein-A-His-tagged yUtp23. Bound material was eluted under denaturing conditions, separated by SDS-PAGE and visualized by Coomassie staining. A total of 10% of the input material was loaded. (**C**) Immobilized GST-tagged yeast yUtp23 or free GST was incubated with proteins generated by *in vitro* translation in the presence of [^35^S] methionine. Bound material was treated as in panel B and analyzed by a PhosphorImager. 2% of the input material was loaded. **D** EMSA showing the binding of GST-yRrp7 (170, 1700 and 3300 nM) or GST (3300 nM) to *in vitro* transcribed radiolabeled snR30 (top) or human U17 (bottom) RNA. RNA and RNP complexes were analyzed as in Figure 2B. (**E**) Recombinant GST-tagged human hUTP23 or free GST was immobilized on glutathione sepharose and incubated with N-terminally His-tagged hUTP24, hRRP7 or hNHP2. Bound material was eluted under denaturing conditions, separated by SDS-PAGE and transferred to nitrocellulose membrane. Proteins were analyzed by immunoblotting using antibodies specific for the His-tag (upper panels) or the GST-tag (lower panel), respectively. Double asterisks: C-terminally truncated forms of the His-tagged hRRP7 protein. The single asterisk denotes a non-specific protein recognizedby the anti-GST antibody (lower panel). Left panel (hUTP24): 10% of the input material was loaded. Right panel (hRRP7, hNHP2): 5% of the input material was loaded. (**F**) Immobilized GST-tagged human hUTP23 or free GST was incubated with hROK1/DDX52 protein generated by *in vitro* translation in the presence of [^35^S] methionine. Bound material was treated as in panel C and analyzed by a PhosphorImager. One percent of the input material was loaded. Asterisk: non-specific protein recognized by the anti-GST antibody.

In recent years, several *in vivo* RNA-protein crosslinking studies in yeast have highlighted the 18S ES6 region as a binding platform for early acting SSU synthesis factors, which contact the 18S rRNA in close proximity to the described yUtp23 crosslinking sites (Figure 3A). In the case of the PIN domain endonuclease yUtp24 (7) and the RNA helicase yDhr1 (31), binding to the ES6 region only represented a secondary pre-rRNA contact, while their main crosslinking sites were located within the pseudoknot region. Consistent with this, snoRNA crosslinks for both proteins were mainly mapped to U3, which forms extensive base-pairing interactions with the central pseudoknot. Two other factors, the RNA helicase yRok1 (20) and the RNA-binding protein yRrp7 (21), were almost exclusively crosslinked to the ES6 region. While both proteins are functionally linked to snR30, yRok1 was shown to crosslink to the SSU-associated snoRNAs snR30, U3 and U14, and, to a lesser extent, to snR10, whereas yRrp7 exhibited a strong association with snR10. Lastly, the CTD domain of the pre-ribosomal ‘compaction factor’ yRrp5 also crosslinked to the ES6 region and analysis of full-length yRrp5 revealed hits to all SSU-associated snoRNAs (U3, snR30, snR10 and U14) (22). Many of these SSU synthesis factors are conserved in the human system and are therefore predicted to engage in similar interactions at the human ES6 region or with the U17 snoRNA, the human snR30 counterpart, but their exact contact sites or interaction partners are not known.

To better understand the role of yUtp23/hUTP23 within this ‘binding platform’ for ribosome biogenesis factors in yeast and humans, we established an *in vitro* system using recombinant proteins (Supplementary Figures S2 and S5) and [^35^S] *in vitro* translated proteins toanalyze protein–protein interactions between ES6-interacting factors (Figure 3, panels B, C, E and F). In these experiments, yeast or human recombinant GST-tagged proteins or free GST were immobilized on glutathione sepharose, incubated with His-tagged recombinant or *in vitro* translated proteins and washed repeatedly to remove non-specifically bound factors. Retained material was eluted under denaturing conditions, separated by SDS-PAGE and visualized by Coomassie staining (panel B), PhosphorImaging (panels C and F) or immunoblotting (using anti-His or anti GST tag antibodies, panel E), respectively.

We firstly found that yeast yUtp23 interacted with itself (panels B and C), suggesting that, similar to other PIN domain proteins (32), it might act as a multimer. Interestingly, we also detected strong interactions between yUtp23 and the PIN domain endonuclease yUtp24 (panel B), which has recently been reported to be directly responsible for the A1 and A2 site cleavages (6,7). This raises the exciting possibility that yUtp23 might act as a recruitment factor or, alternatively, a chaperone for yUtp24, to modulate yUtp24 catalytic activity until it is correctly positioned within the SSU processome. We did not, however, observe interactions between yUtp23 and yDhr1 (panel C) or yRrp5 (not shown).

yUtp23 also specifically interacted with yRrp7 (panel B) and yRok1 (panel C). In yeast, snR30 is required for the stable association of yRrp7 to pre-ribosomes but not vice versa (21). We therefore speculate that yRrp7 contacts the pre-rRNA either during snR30 base-pairing or after snR30 action (see discussion). Consistent with the hypothesis that yRrp7 might associate with the 18S sequence in the absence of snR30, the top snoRNA in the yRrp7 CRAC data set was indeed snR10, and not snR30 (21), and we were unable to observe a direct interaction between recombinant GST-yRrp7 and *in vitro* transcribed snR30 (Figure 3D, upper panel), or, in fact, snR10 (data not shown). Curiously, GST-yRrp7 strongly interacted with U17 (Figure 3D, lower panel), but yRrp7 had also previously been reported to interact with another, functionally unrelated box H/ACA snoRNA, snR5, *in vitro* (21). It is therefore unclear if the observed yRrp7/U17 interaction is biologically relevant. Given that the U17 and snR5 snoRNAs both lack an extended internal hairpin (Supplementary Figure S4D and data not shown), it is also possible that the snR30-specific internal hairpin might interfere with yRrp7 binding to snR30 in our *in vitro* assay.

We further assessed whether yUtp23 directly associates with other known snR30 binding factors. We detected a reproducible interaction between yUtp23 and the nucleolar protein yKri1 (Figure 3, panel C), which, like yUtp23, requires snR30 for pre-ribosomal recruitment (15). Furthermore, we also found that yUtp23 directly interacted with yNhp2, one of the four core box H/ACA proteins, while no interaction was detected with the U3-specific yRrp9 protein. It is likely that the observed protein–protein interaction between yUtp23 and yNhp2 contributes to yUtp23 binding to the snR30 snoRNP. This might explain why the internal hairpin in snR30, which is important for binding of yUtp23 to snR30 *in vitro* (Figure 2F), is not essential for survival *in vivo* (18).

Finally, we also tested human homologues of key ES6-binding factors (hUTP24, hROK1/DDX52 and hRRP7) as well as the core box H/ACA protein hNHP2, for their ability to interact with recombinant GST-tagged human hUTP23 (panels E and F, respectively). Importantly, positive interactions detected in yeast (panels B and C) were also detected in the human system. Human hUTP23 interacted with hUTP24, hRRP7, hNHP2 and hROK1/DDX52. This indicates that key protein–protein interactions at the 18S ES6 rRNA region are evolutionarily conserved.

In conclusion, the observed direct interactions between yUtp23/hUTP23 and factors that also bind to the ES6 region or the snR30/U17 snoRNAs, in combination with the positioning of their crosslink sites on the individual RNAs, provide important insights into their spatial and temporal association within the yeast and human SSU processome (see discussion).

### An intact PIN domain and Zinc finger in human hUTP23 are both required for 18S rRNA maturation

**Figure 4.**
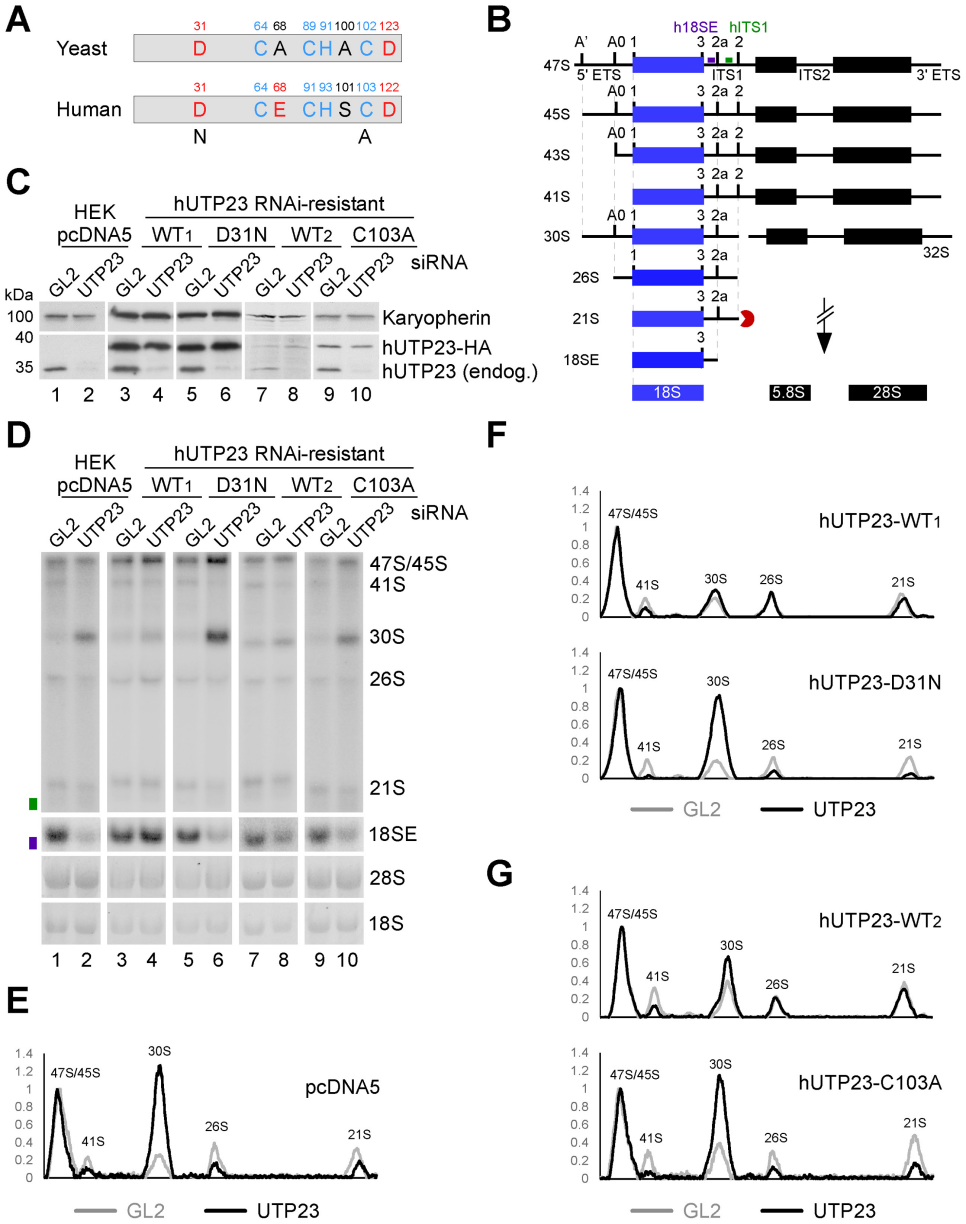
Intact PIN domain and Zinc finger motifs in human hUTP23 are essential for 18S maturation. (**A**) Cartoon depiction of the PIN domain and CCHC Zinc finger motifs in yeast yUtp23 and human hUTP23. Acidic and non-acidic residues in the proposed catalytic centre of the PIN domain are marked in red and black, respectively. Blue: conserved Zinc finger residues. Point mutations generated in the PIN domain (D31N) or the Zinc finger (C103A) of hUTP23 are indicated. (**B**) Schematic representation of the key steps in human ribosome biogenesis. Radiolabeled probes used for northern blotting (h18SE; purple and hITS1; green) are marked above the 47S precursor. ETS: external transcribed spacer; ITS: internal transcribed spacer. (**C**) HEK293 cells were stably transfected with a control plasmid (pcDNA5) or constructs encoding wild type (WT) or mutant forms of HA-tagged hUTP23 (D31N, C103A) and treated with tetracycline to induce protein expression. The hUTP23 coding sequence was modified to render the expressed mRNA resistant to the hUTP23 siRNA. Protein extracted from control cells (GL2), or those depleted of endogenous hUTP23 (UTP23) by RNAi, was separated by SDS-PAGE and transferred to nitrocellulose membrane. Protein levels were analyzed by immunoblotting using antibodies specific for hUTP23 (lower panel) or Karyopherin (upper panel) as loading control. (**D**) RNA from stably transfected and RNAi-treated HEK293 cells as shown in panel C was analyzed by northern blotting using probes hybridizing to the 5΄ end of ITS1 (h18SE, purple rectangle) or downstream of 2a (hITS1, green rectangle). Pre-rRNAs were detected using a PhosphorImager and total rRNA (28S/18S) was visualized by ethidium bromide (EtBr) staining. RNA species are labeled on the right. (**E–G**) RNA levels from panel D were normalized to the 47S/45S pre-rRNAs and plotted for each GL2 (gray) or hUTP23 (black) knockdown. The identity of each peak is indicated.

In budding yeast, yUtp23 contains only two (D31 and D123) of the four possible acidic residues that make up the characteristic PIN domain endonuclease catalytic center (Figure 4A and Supplementary Figure S6), and mutational analyses of these two residues strongly suggested a non-enzymatic role for yUtp23 within the yeast SSU processome (4,14). The PIN domain of human hUTP23, on the other hand, contains conserved acidic residues at three positions (D31, E68 and D122) and it was shown for the PIN domain of the NMD endonuclease SMG6, that a triad of acidic residues is sufficient for catalytic activity (33). Both human and yeast Utp23 proteins also share a conserved CCHC Zinc finger motif with a predicted role in nucleic acid binding. Single point mutations in the Zinc finger of yeast yUtp23 are lethal (14), but the essential role of this motif has not yet been determined.

RNAi-mediated knockdown of human hUTP23 results in 30S pre-rRNA accumulation, indicating that the presence of hUTP23 is required for the A0, 1 and 2a pre-rRNA cleavages (12) (Figure 4B). The PIN endonuclease hUTP24 is responsible for cleavages at sites 1 and 2a (6,7), but the identity of the enzyme that cleaves at site A0 remains unknown. To investigate a potential catalytic function of human hUTP23 in site A0 cleavage, and to understand the role of its conserved Zinc finger motif, we established an RNAi-rescue system for hUTP23 in HEK293 cells. For this, cells were stably transfected with an empty control vector (pcDNA5) or plasmids encoding C-terminally 2xHA-tagged hUTP23 carrying silent mutations in the hUTP23 open reading frame rendering it resistant to RNAi-knockdown when the endogenous hUTP23 mRNA is targeted. Cells expressing hUTP23 WT or mutant hUTP23 proteins with a single catalytic site mutation in the PIN domain (D31N) or a point mutation in the Zinc finger motif (C103A) were compared. Using a titratable *TET* promoter, conditions were established to express WT and mutant proteins at equivalent levels (Figure 4C). WT and mutant proteins showed some variation in their response to induction by tetracycline and it was therefore difficult to titrate the expression of all HA-tagged proteins to a level equivalent to that of the endogenous protein. To address this, two separate WT samples are presented in Figure 4C, with expression levels that are directly comparable to either the D31N (WT1, compare lanes 3–6) or the C103A (WT2, compare lanes 7–10) mutant, respectively. Cells were transfected with either a siRNA specifically targeting endogenous hUTP23 or a control siRNA targeting firefly luciferase (GL2) (34). After siRNA treatment for 72 h, the expression of endogenous and HA-tagged hUTP23 proteins was analyzed by immunoblotting. The hUTP23-specific siRNA significantly reduced endogenous protein levels, whereas the RNAi-resistant HA-tagged hUTP23 proteins were unaffected (Figure 4C).

Total RNA was extracted from RNAi-treated cells and pre-rRNA processing was analyzed by Northern hybridization using probes complementary to the 5΄ end of ITS1 (‘h18SE’) or downstream of the 2a cleavage site (‘hITS1’) (Figure 4, panels B and D–G). Depletion of hUTP23 resulted in a significant accumulation of the 30S pre-rRNA, indicative of strongly reduced early cleavages at the A0, 1 and 2a sites, and consequently, a substantial reduction in 18SE levels as previously reported (Figure 4, panels D and E) (12). A 5΄-extended 30S precursor (referred to as 30SL5’ (12) or 34S (35)), did not accumulate, suggesting that cleavage at A’ was not affected (Figure 4D, compare lanes 1 and 2). In cells expressing moderately high levels of wild type HA-tagged hUTP23 (WT1), knockdown of endogenous hUTP23 had no effect on either 18SE or 30S pre-rRNA levels (compare lanes 3 and 4, see panel F for quantification), demonstrating that HA-tagged hUTP23 can functionally replace the endogenous protein in our system. While expression of lower levels of the WT protein (WT2; note that levels are slightly lower than the endogenous protein) did not rescue the processing phenotype to the same extent, a significant reduction of the 30S levels compared to the hUTP23 depletion phenotype was still observed (compare lanes 8 and 2, see panels G and E for quantification).

Surprisingly, expression of either the PIN (D31N, Figure 4D, lane 6) or the Zinc finger (C103A, lane 10) hUTP23 mutants, after hUTP23 knockdown, resulted in strong 30S accumulation compared to their respective WT control (see panels F and G for quantification), a phenotype identical to hUTP23 depletion in the absence of exogenous protein (lane 2). This indicates that the putative active site in the PIN domain of hUTP23 and the integrity of the conserved Zinc finger are both important for hUTP23 function in pre-rRNA processing.

We next investigated the reason(s) why the PIN and Zinc finger mutations impeded hUTP23 function. The significant accumulation of the 30S pre-rRNA caused by expression of the hUTP23 D31N PIN mutant *in vivo* (Figure 4D, lane 6) would be readily explained by a loss of enzymatic activity at site A0. Since site A0, 1 and 2a cleavages are linked, a defect in A0 cleavage would also lead to defects in the cleavages at sites 1 and 2a, which is fitting with the substantial increase in 30S pre-rRNA levels seen upon expression of the hUTP23 D31N PIN mutant. We therefore tested whether recombinant hUTP23 (see Supplementary Figure S5A) would cleave an RNA substrate containing human site A0. However, attempts to demonstrate nuclease activity were so far unsuccessful (data not shown and see discussion).

**Figure 5.**
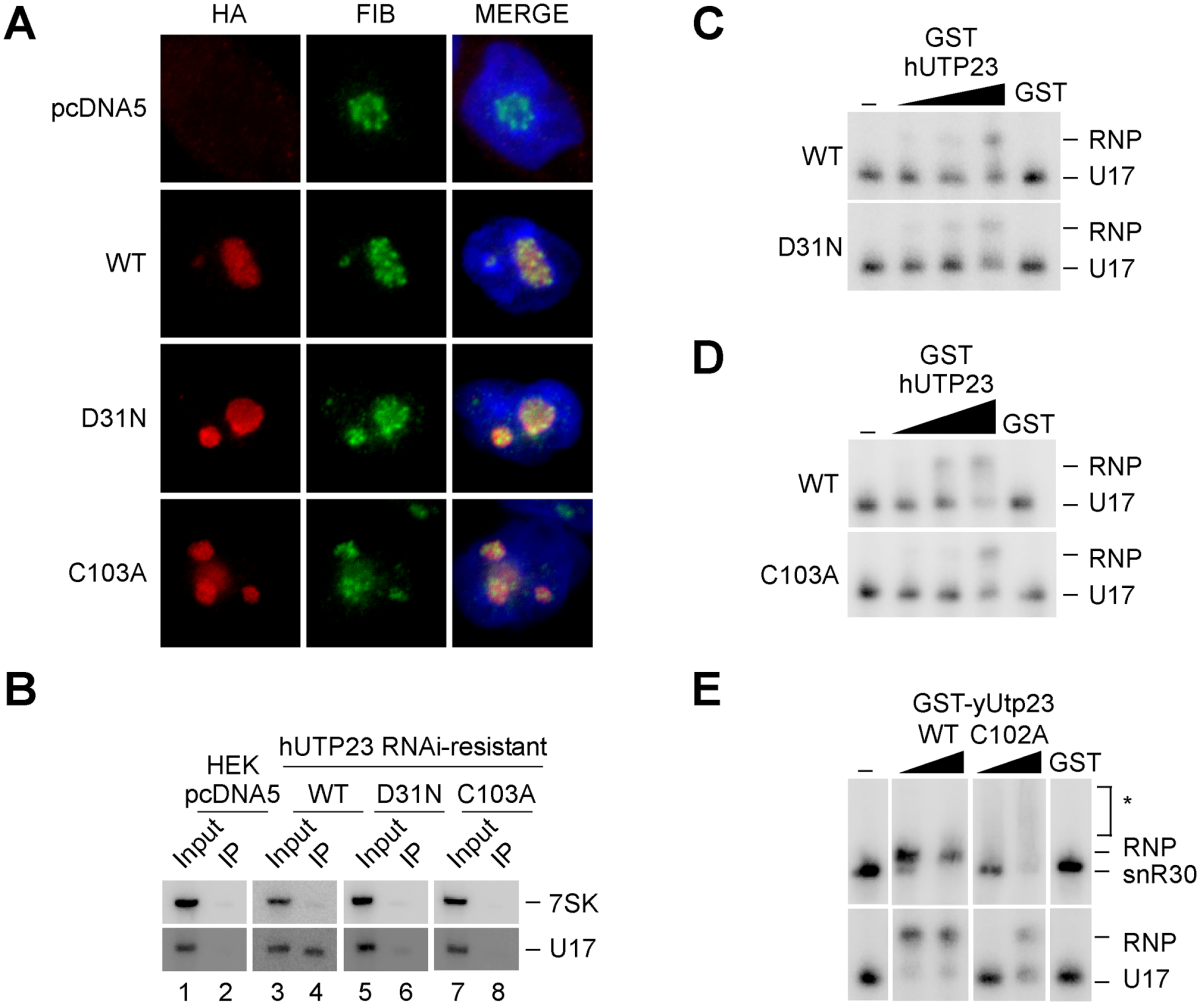
The hUTP23 Zinc finger mutant exhibits typical nucleolar localization, but impaired *in vivo* and *in vitro* binding to U17. (**A**) Cells expressing WT or mutant forms of HA-tagged hUTP23 (D31N, C103A) or no HA-tagged protein (pcDNA5) were harvested and processed for immunofluorescence microscopy using an anti-HA antibody (red). Cells were counterstained with an antibody against endogenous fibrillarin (FIB, green) as a nucleolar marker and DAPI (blue) to highlight the nucleus. From left to right: Immunofluorescence signals for the anti-HA and anti-fibrillarin antibodies and the merged image. (**B**) Soluble lysates from cells, as shown in panel A, were subjected to immunoprecipitation using an anti-HA antibody. Co-precipitated RNA was extracted and separated by denaturing acrylamide electrophoresis, analyzed by northern blotting using radiolabeled probes against the U17 snoRNA and 7SK as a loading control and visualized using a PhosphorImager. A total of 1.25% of the input material was loaded. (**C**) EMSA showing the binding of recombinant wild type (WT, top panel) or D31N PIN mutant (bottom panel) human GST-hUTP23 proteins (450, 900 and 1800 nM) or GST (1800 nM) to *in vitro* transcribed radiolabeled U17 snoRNA. RNA and RNP complexes were analyzed as in Figure 2B. (**D**) Binding of WT (top panel) or the C103A Zn finger mutant (bottom panel) human GST-hUTP23 proteins (750, 1500 and 3000 nM) or free GST (3000 nM) to the U17 snoRNA. Analysis as described in panel C. (**E**) Binding of recombinant WT and Zinc finger mutant (C102A) yeast GST-yUtp23 proteins (250 and 2500 nM) or GST (2500 nM) to snR30 (top) or U17 (bottom) RNAs. Asterisks: unresolved C102A mutant-snR30 complexes, likely due to non-stable protein–RNA interactions. Analysis as described in panel C.

Immunofluorescence experiments using cells expressing HA-tagged proteins demonstrated that, in contrast to fibrillarin, which is exclusively found in the dense fibrillar component (29), wild-type hUTP23 is localized throughout the nucleolus (Figure 5A). Similar to the wild type protein, the mutant hUTP23 proteins were also found throughout the nucleolus. However, immunoprecipitation experiments revealed that only the WT hUTP23 protein was stably associated with the human homologue of snR30, the U17 snoRNA (Figure 5B, lane 4). The PIN mutant exhibited considerably weaker, but still detectable association with the U17 snoRNA (see lane 6), whereas the C103A Zinc finger mutant did not co-precipitate the U17 snoRNA above background (lane 8). Consistent with our yeast data (Figure 2), recombinant WT hUTP23 directly bound to *in vitro* transcribed U17 snoRNA (Figure 5C and 5D, upper panels). The D31N PIN mutant associated with U17 with similar affinity (Figure 5C, lower panel), whereas the C103A Zinc finger mutant exhibited significantly reduced binding (Figure 5D, lower panel). Interestingly, the same result was observed when we analyzed the binding of WT recombinant yeast yUtp23 and a C102A Zinc finger mutant (Supplementary Figure S5B) to either yeast snR30 or human U17 (Figure 5E). The WT yUtp23 protein efficiently bound both U17 and snR30, but in contrast, the C102A Zinc finger mutant showed significantly reduced binding to both snoRNAs at low protein concentration. In the presence of high levels of the C102A mutant, about half of the U17 substrate was still bound compared to the wild type protein, whereas resolvable complexes of bound snR30 or free RNA could not be detected. This result suggests non-stoichiometric and likely non-specific protein-snR30 interactions when the mutant protein is present at high concentration in the assay. The *in vitro* RNA binding results indicate that the severe growth defect in yeast (14) and the pre-rRNA processing defect in humans (Figure 4), seen upon expression of the yUtp23/hUTP23 Zinc finger mutants, are likely due to a defect in yUtp23/hUTP23 binding to the snR30 and U17 snoRNAs, respectively.

Taken together, our data indicate that an intact PIN domain in hUTP23 and efficient binding of hUTP23 to the U17 snoRNP are both required for 18S maturation in humans.

## DISCUSSION

Here, we present data suggesting that the yeast yUtp23-snR30 and human hUTP23-U17 complexes may act as hubs to coordinate the binding and release of factors at the 18S ES6 region. We also show that the PIN domain active site amino acid, D31, is needed for pre-rRNA processing suggesting that hUTP23 may actually be an endonuclease.

**Figure 6.**
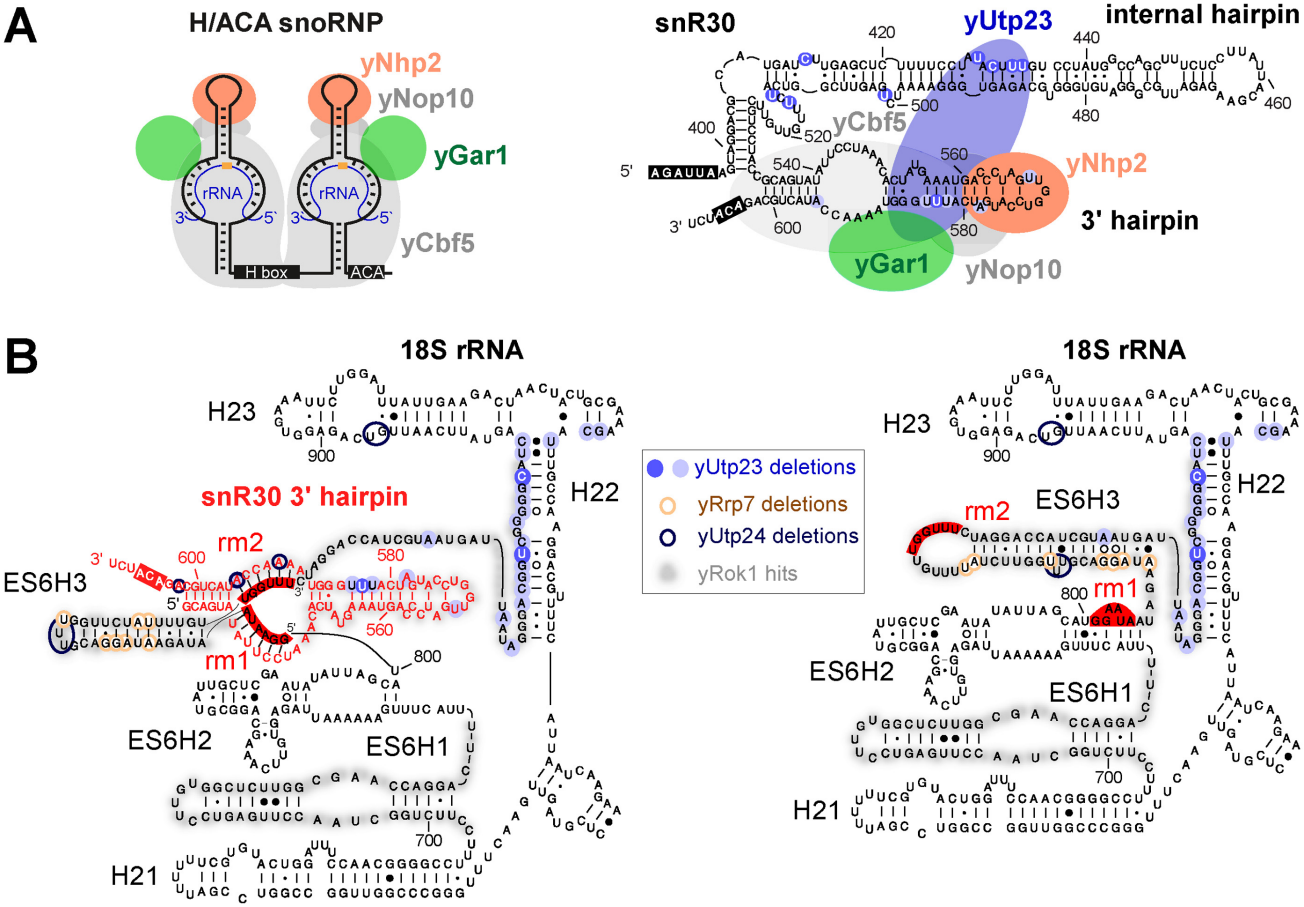
Yeast yUtp23 is central to the integration of ES6 binding factors and snR30 release. (**A**) Left panel: Cartoon of a typical modification H/ACA box yeast snoRNP depicting the positions of the core H/ACA snoRNP proteins yCbf5, yGar1, yNop10 and yNhp2. H and ACA boxes are shown in black. Orange circle: target nucleotide for pseudouridylation in the rRNA (blue). Right panel: Cartoon of the 3΄ terminal portion of yeast snR30 with the predicted positions of yUtp23 and core H/ACA snoRNP proteins. yUtp23 microdeletion sites in the internal hairpin and 3΄ hairpin are represented as blue circles, shades represent peak height as in Figure 2C. The H and ACA boxes are shown in black. (**B**) Predicted structure of the yeast 18S ES6 region during snR30 base-pairing (left panel) and in the mature rRNA after snR30 release (right panel). Precise crosslinking sites of yUtp23 (blue circles, shades indicate peak height as in Figures 1 and 2), yUtp24 (dark blue) and yRrp7 (brown), and yRok1 crosslinking regions (shaded in grey) on the snR30 3΄ hairpin (red) and 18S rRNA sequences (black). Binding sites for snR30 in the ES6 region (rm1 and rm2) and the ACA box are highlighted in red.

Using *in vivo* crosslinking in actively growing yeast cells, we have identified snR30 and the 18S ES6 region as *bona fide* RNA-binding targets for yUtp23 (Figures 1 and 2, and Supplementary Figures S2, S3 and S4). Direct protein-RNA interactions were validated *in vitro* using recombinant proteins and *in vitro* transcribed RNA and we also showed that stable interactions with snR30/U17 required an intact Zinc finger in yUtp23/hUTP23 (Figure 5). The top of the 3΄ hairpin in snR30 was predicted to bind a protein essential for snR30 function in 18S rRNA processing (19) and yUtp23 has been identified, alongside yKri1, as a novel non-core snoRNP protein co-purifying with snR30 (15). Interestingly, we detected a direct protein–protein interaction between yUtp23 and yKri1 (Figure 3C). This result was surprising, given that these proteins have been suggested to associate with snR30 in a mutually exclusive manner (15). It was also shown in the same study that yUtp23, but not yKri1, is essential for snR30 release. The observed direct interaction between both proteins may therefore suggest that they are involved in distinct, but possibly consecutive steps of snR30 function, with yUtp23 playing a more active role in snR30 release. Our *in vivo* crosslinking and *in vitro* RNA binding studies have revealed that yUtp23 primarily contacts the top part of the 3΄ hairpin and the internal hairpin of snR30 (Figures 2 and 6A). Our data further showed that all three hairpins in snR30 are important for efficient binding of yUtp23 *in vitro*. The finding that the internal hairpin is a major snR30 binding site of the essential yUtp23 protein *in vivo* and *in vitro* is surprising given that this region of the snoRNA has been shown to be dispensable for snR30 function *in vivo* (18). Interestingly, we also discovered an evolutionarily conserved interaction between yUtp23/hUTP23 and the H/ACA snoRNP core component, yNhp2/hNHP2 (Figure 3) and yeast yUtp23 was previously reported to interact with another H/ACA snoRNP component, yGar1 (36). We believe that the interactions between yUtp23 and both yNhp2 and yGar1, which are expected to also bind to the upper part of the snR30 3΄ hairpin (Figure 6A), are sufficient to tether yUtp23 on the snR30 snoRNP and thereby compensate for the lack of the internal hairpin *in vivo*. While further experiments are clearly needed to clarify the exact role of yKri1 with respect to snR30 function, our data strongly suggest that yUtp23 is one of the predicted essential snR30-specific protein(s) that bind the 3΄ hairpin in the snoRNA.

*In vivo* protein–RNA crosslinks were also found between yUtp23 and the ES6 region of the 18S rRNA. The specificity of this interaction was validated with an *in vitro* RNA binding experiment (Supplementary Figure S2D). Primer extension analyses of RNAs crosslinked to both yeast yUtp23 and human hUTP23 proteins further revealed that this interaction is evolutionarily conserved (Supplementary Figure S3). Our data therefore show that yUtp23/hUTP23 binds directly to two different RNAs, snR30/U17 and the 18S rRNA ES6 region. Interestingly, the main 18S rRNA interaction site for yUtp23/hUTP23 is in close proximity to the snR30/U17 base-pairing regions in ES6 (rm1 and rm2) and near the known crosslinking sites for yeast yRok1, yRrp7, yDhr1, yUtp24 and yRrp5 (Figure 6B). Furthermore, we could show direct protein–protein interactions between yUtp23 and yRok1, yRrp7 and yUtp24 and their human equivalents hUTP23, hROK1, hRRP7 and hUTP24 (Figure 3). The yUtp23-snR30 and hUTP23-U17 complexes therefore appear to form central hubs within the yeast and human SSU processomes that potentially co-ordinate the binding of factors to, and their subsequent release from, ES6. Indeed, it has already been shown that yUtp23 is needed for snR30 release from the pre-rRNA (15), a process involving the RNA helicase yRok1 and the compaction factor yRrp5 (23,24).

*In vivo* crosslinking studies in yeast revealed that several of the protein contact sites in ES6 overlap (Figure 6B), suggesting sequential and coordinated protein binding. Consistent with this idea, some of the ES6 binding proteins, such as yRrp5, yRrp7 and yUtp24, are still present in the 90S pre-ribosome after snR30 and yUtp23 have left the complex (37). Notably, snR30 is required for stable association of yRrp7 with pre-ribosomes, but not vice versa (21). Given the dramatic change in the structure of the yRrp7 binding site in ES6 seen before and after snR30 release (Figure 6B, compare left and right panels), it is unlikely that yRrp7 binds to both structures. yRrp7 is present in 90S pre-ribosomes lacking snR30 and, within this complex, probably still bound to the pre-rRNA after snR30 release. We therefore hypothesize that yRrp7 recognizes the post-snR30 ES6 structure. Supporting this idea, yRrp7 mainly crosslinked to snR10 instead of snR30 (21) and we did not detect stable *in vitro* interactions between yRrp7 and snR30 (Figure 3D). yUtp23 may therefore, together with snR30, function as a platform to recruit yRrp7 to the pre-ribosome through protein–protein interactions. Subsequently, the yRrp7 binding site may then be generated by the yUtp23-dependent release of snR30 and the re-structuring of ES6. Protein–protein interactions between yUtp23 and yRok1 suggest a similar scenario, where yUtp23 could contribute to the role of yRok1 in releasing snR30 from the pre-ribosome. In this context, we were surprised to observe an *in vitro* interaction between yeast yRrp7 and human U17 (Figure 3D). However, future studies in human cells will be needed to clarify whether hRRP7 and U17 interact *in vivo* and whether this is biologically relevant.

Using an RNAi-rescue system, we have also analyzed the function of human hUTP23. Knockdown of hUTP23 inhibits cleavages at sites A0, 1 and 2a (12). These defects were rescued by re-expression of the WT protein, but not hUTP23 containing point mutations in either the Zinc finger or the PIN domain (Figure 4), although WT and both mutant proteins localized to the nucleolus as expected (Figure 5). We observed that the hUTP23 Zinc finger mutant (C103A) showed strongly reduced association with the U17 snoRNA *in vitro* and *in vivo*. The equivalent point mutation in yeast yUtp23 (C102A) also impaired *in vitro* interactions with snR30 (Figure 5) and caused lethality (14). In yeast, snR30 is needed for correct positioning of yUtp23 within the SSU processome to enable yUtp23 binding to the 35S pre-rRNA (15). Our comparative analyses therefore indicate that a direct protein–RNA interaction between yUtp23/hUTP23 and snR30/U17 is evolutionarily conserved and is dependent on the integrity of the Zinc finger.

An intact PIN domain, on the other hand, is not essential for yUtp23 function in yeast (4,14). In humans, however, the PIN domain mutant (D31N) did not rescue hUTP23 function despite correct nucleolarlocalization. Binding of the recombinant mutant protein to the U17 snoRNA was not affected *in vitro*, indicating that there is no major defect in protein folding caused by the single point mutation in the PIN domain. We did, however, notice significantly decreased co-precipitation of U17 with the hUTP23 PIN mutant *in vivo*. It is therefore possible that the D31N mutation might interfere with other essential hUTP23 interactions within the human pre-40S particle that are needed for U17 association. In yeast and other fungi, two of the four PIN domain active site amino acids are present in yUtp23 (Supplementary Figure S6), however, in metazoan and plant UTP23, three of the key active-site amino acids are conserved. In some PIN-domain proteins, such as SMG6, this is sufficient for nuclease activity (33). Our attempts to detect nuclease activity in human hUTP23, using a variety of human pre-ribosomal RNA substrates containing site A0 have so far been unsuccessful. While the current lack of *in vitro* cleavage data could indicate a non-catalytic role for hUTP23 in human ribosome biogenesis, the strong pre-rRNA processing defects observed upon expression of the PIN mutant *in vivo* make it likely that instead the right *in vitro* substrate has not yet been identified. In addition, other protein factors may be needed for correct RNA folding *in vitro* and/or activation of hUTP23. Indeed, the human PIN domain protein hNOB1 was recently shown to require a co-factor, hCINAP1, for cleavage at site 3 *in vitro* (11). Future experiments will reveal whether hUTP23 is an active enzyme and whether a co-factor(s) is required for its activity.

## ACCESSION NUMBERS

GEO database (http://www.ncbi.nlm.nih.gov/geo/): identifier GSE87238.

## Supplementary Material

Supplementary Data

## References

[1] Henras A.K., Plisson-Chastang C., O’Donohue M.F., Chakraborty A., Gleizes P.E. An overview of pre-ribosomal RNA processing in eukaryotes. Wiley Interdiscip. Rev. RNA. 2015; 6:225–242. 25346433 10.1002/wrna.1269 PMC4361047

[2] Phipps K.R., Charette J., Baserga S.J. The small subunit processome in ribosome biogenesis-progress and prospects. Wiley Interdiscip. Rev. RNA. 2011; 2:1–21. 21318072 10.1002/wrna.57 PMC3035417

[3] Watkins N.J., Bohnsack M.T. The box C/D and H/ACA snoRNPs: key players in the modification, processing and the dynamic folding of ribosomal RNA. Wiley Interdiscip. Rev. RNA. 2012; 3:397–414. 22065625 10.1002/wrna.117

[4] Bleichert F., Granneman S., Osheim Y.N., Beyer A.L., Baserga S.J. The PINc domain protein Utp24, a putative nuclease, is required for the early cleavage steps in 18S rRNA maturation. Proc. Natl. Acad. Sci. U.S.A. 2006; 103:9464–9469. 16769905 10.1073/pnas.0603673103 PMC1480430

[5] Sloan K.E., Mattijssen S., Lebaron S., Tollervey D., Pruijn G.J., Watkins N.J. Both endonucleolytic and exonucleolytic cleavage mediate ITS1 removal during human ribosomal RNA processing. J. Cell Biol. 2013; 200:577–588. 23439679 10.1083/jcb.201207131 PMC3587827

[6] Tomecki R., Labno A., Drazkowska K., Cysewski D., Dziembowski A. hUTP24 is essential for processing of the human rRNA precursor at site A1, but not at site A0. RNA Biol. 2015; 12:1010–1029. 26237581 10.1080/15476286.2015.1073437 PMC4615547

[7] Wells G.R., Weichmann F., Colvin D., Sloan K.E., Kudla G., Tollervey D., Watkins N.J., Schneider C. The PIN domain endonuclease Utp24 cleaves pre-ribosomal RNA at two coupled sites in yeast and humans. Nucleic Acids Res. 2016; 44:5399–5409. 27034467 10.1093/nar/gkw213 PMC4914098

[8] Fatica A., Oeffinger M., Dlakic M., Tollervey D. Nob1p is required for cleavage of the 3΄ end of 18S rRNA. Mol. Cell. Biol. 2003; 23:1798–1807. 12588997 10.1128/MCB.23.5.1798-1807.2003 PMC151717

[9] Pertschy B., Schneider C., Gnadig M., Schafer T., Tollervey D., Hurt E. RNA helicase Prp43 and its co-factor Pfa1 promote 20 to 18 S rRNA processing catalyzed by the endonuclease Nob1. J. Biol. Chem. 2009; 284:35079–35091. 19801658 10.1074/jbc.M109.040774 PMC2787369

[10] Lebaron S., Schneider C., van Nues R.W., Swiatkowska A., Walsh D., Bottcher B., Granneman S., Watkins N.J., Tollervey D. Proofreading of pre-40S ribosome maturation by a translation initiation factor and 60S subunits. Nat. Struct. Mol. Biol. 2012; 19:744–753. 22751017 10.1038/nsmb.2308 PMC3654374

[11] Bai D., Zhang J., Li T., Hang R., Liu Y., Tian Y., Huang D., Qu L., Cao X., Ji J. The ATPase hCINAP regulates 18S rRNA processing and is essential for embryogenesis and tumour growth. Nat. Commun. 2016; 7:12310. 27477389 10.1038/ncomms12310 PMC4974663

[12] Sloan K.E., Bohnsack M.T., Schneider C., Watkins N.J. The roles of SSU processome components and surveillance factors in the initial processing of human ribosomal RNA. RNA. 2014; 20:540–550. 24550520 10.1261/rna.043471.113 PMC3964915

[13] Wang M., Anikin L., Pestov D.G. Two orthogonal cleavages separate subunit RNAs in mouse ribosome biogenesis. Nucleic Acids Res. 2014; 42:11180–11191. 25190460 10.1093/nar/gku787 PMC4176171

[14] Lu J., Sun M., Ye K. Structural and functional analysis of Utp23, a yeast ribosome synthesis factor with degenerate PIN domain. RNA. 2013; 19:1815–1824. 24152547 10.1261/rna.040808.113 PMC3860261

[15] Hoareau-Aveilla C., Fayet-Lebaron E., Jady B.E., Henras A.K., Kiss T. Utp23p is required for dissociation of snR30 small nucleolar RNP from preribosomal particles. Nucleic Acids Res. 2012; 40:3641–3652. 22180534 10.1093/nar/gkr1213 PMC3333846

[16] Morrissey J.P., Tollervey D. Yeast snR30 is a small nucleolar RNA required for 18S rRNA synthesis. Mol. Cell. Biol. 1993; 13:2469–2477. 8455623 10.1128/mcb.13.4.2469-2477.1993 PMC359567

[17] Lemay V., Hossain A., Osheim Y.N., Beyer A.L., Dragon F. Identification of novel proteins associated with yeast snR30 small nucleolar RNA. Nucleic Acids Res. 2011; 39:9659–9670. 21893585 10.1093/nar/gkr659 PMC3239182

[18] Atzorn V., Fragapane P., Kiss T. U17/snR30 is a ubiquitous snoRNA with two conserved sequence motifs essential for 18S rRNA production. Mol. Cell. Biol. 2004; 24:1769–1778. 14749391 10.1128/MCB.24.4.1769-1778.2004 PMC344193

[19] Fayet-Lebaron E., Atzorn V., Henry Y., Kiss T. 18S rRNA processing requires base pairings of snR30 H/ACA snoRNA to eukaryote-specific 18S sequences. EMBO J. 2009; 28:1260–1270. 19322192 10.1038/emboj.2009.79 PMC2664660

[20] Martin R., Hackert P., Ruprecht M., Simm S., Bruning L., Mirus O., Sloan K.E., Kudla G., Schleiff E., Bohnsack M.T. A pre-ribosomal RNA interaction network involving snoRNAs and the Rok1 helicase. RNA. 2014; 20:1173–1182. 24947498 10.1261/rna.044669.114 PMC4105744

[21] Lin J., Lu J., Feng Y., Sun M., Ye K. An RNA-binding complex involved in ribosome biogenesis contains a protein with homology to tRNA CCA-adding enzyme. PLoS Biol. 2013; 11:e1001669. 24130456 10.1371/journal.pbio.1001669 PMC3794860

[22] Lebaron S., Segerstolpe A., French S.L., Dudnakova T., de Lima Alves F., Granneman S., Rappsilber J., Beyer A.L., Wieslander L., Tollervey D. Rrp5 binding at multiple sites coordinates pre-rRNA processing and assembly. Mol. Cell. 2013; 52:707–719. 24239293 10.1016/j.molcel.2013.10.017 PMC3991325

[23] Bohnsack M.T., Kos M., Tollervey D. Quantitative analysis of snoRNA association with pre-ribosomes and release of snR30 by Rok1 helicase. EMBO Rep. 2008; 9:1230–1236. 18833290 10.1038/embor.2008.184 PMC2570499

[24] Khoshnevis S., Askenasy I., Johnson M.C., Dattolo M.D., Young-Erdos C.L., Stroupe M.E., Karbstein K. The DEAD-box protein Rok1 orchestrates 40S and 60S ribosome assembly by promoting the release of Rrp5 from Pre-40S ribosomes to allow for 60S maturation. PLoS Biol. 2016; 14:e1002480. 27280440 10.1371/journal.pbio.1002480 PMC4900678

[25] Granneman S., Petfalski E., Tollervey D. A cluster of ribosome synthesis factors regulate pre-rRNA folding and 5.8S rRNA maturation by the Rat1 exonuclease. EMBO J. 2011; 30:4006–4019. 21811236 10.1038/emboj.2011.256 PMC3209772

[26] Granneman S., Kudla G., Petfalski E., Tollervey D. Identification of protein binding sites on U3 snoRNA and pre-rRNA by UV cross-linking and high-throughput analysis of cDNAs. Proc. Natl. Acad. Sci. U.S.A. 2009; 106:9613–9618. 19482942 10.1073/pnas.0901997106 PMC2688437

[27] Wlotzka W., Kudla G., Granneman S., Tollervey D. The nuclear RNA polymerase II surveillance system targets polymerase III transcripts. EMBO J. 2011; 30:1790–1803. 21460797 10.1038/emboj.2011.97 PMC3102002

[28] Schneider C., Anderson J.T., Tollervey D. The exosome subunit Rrp44 plays a direct role in RNA substrate recognition. Mol. Cell. 2007; 27:324–331. 17643380 10.1016/j.molcel.2007.06.006 PMC7610968

[29] Turner A.J., Knox A.A., Watkins N.J. Nucleolar disruption leads to the spatial separation of key 18S rRNA processing factors. RNA Biol. 2012; 9:175–186. 22418842 10.4161/rna.18811

[30] Schneider C., Kudla G., Wlotzka W., Tuck A., Tollervey D. Transcriptome-wide analysis of exosome targets. Mol. Cell. 2012; 48:422–433. 23000172 10.1016/j.molcel.2012.08.013 PMC3526797

[31] Sardana R., Liu X., Granneman S., Zhu J., Gill M., Papoulas O., Marcotte E.M., Tollervey D., Correll C.C., Johnson A.W. The DEAH-box helicase Dhr1 dissociates U3 from the pre-rRNA to promote formation of the central pseudoknot. PLoS Biol. 2015; 13:e1002083. 25710520 10.1371/journal.pbio.1002083 PMC4340053

[32] Lamanna A.C., Karbstein K. Nob1 binds the single-stranded cleavage site D at the 3΄-end of 18S rRNA with its PIN domain. Proc. Natl. Acad. Sci. U.S.A. 2009; 106:14259–14264. 19706509 10.1073/pnas.0905403106 PMC2732849

[33] Glavan F., Behm-Ansmant I., Izaurralde E., Conti E. Structures of the PIN domains of SMG6 and SMG5 reveal a nuclease within the mRNA surveillance complex. EMBO J. 2006; 25:5117–5125. 17053788 10.1038/sj.emboj.7601377 PMC1630413

[34] Elbashir S.M., Harborth J., Weber K., Tuschl T. Analysis of gene function in somatic mammalian cells using small interfering RNAs. Methods. 2002; 26:199–213. 12054897 10.1016/S1046-2023(02)00023-3

[35] Tafforeau L., Zorbas C., Langhendries J.L., Mullineux S.T., Stamatopoulou V., Mullier R., Wacheul L., Lafontaine D.L. The complexity of human ribosome biogenesis revealed by systematic nucleolar screening of Pre-rRNA processing factors. Mol. Cell. 2013; 51:539–551. 23973377 10.1016/j.molcel.2013.08.011

[36] Tarassov K., Messier V., Landry C.R., Radinovic S., Serna Molina M.M., Shames I., Malitskaya Y., Vogel J., Bussey H., Michnick S.W. An in vivo map of the yeast protein interactome. Science. 2008; 320:1465–1470. 18467557 10.1126/science.1153878

[37] Kornprobst M., Turk M., Kellner N., Cheng J., Flemming D., Kos-Braun I., Kos M., Thoms M., Berninghausen O., Beckmann R. Architecture of the 90S Pre-ribosome: A Structural View on the Birth of the Eukaryotic Ribosome. Cell. 2016; 166:380–393. 27419870 10.1016/j.cell.2016.06.014

