# Supplementary material for: The ribosome biogenesis factor yUtp23/hUTP23 coordinates key interactions in the yeast and human pre-40S particle and hUTP23 contains an essential PIN domain
Source: Nucleic Acids Res. 2017 Jan 13;45(8):4796–809. doi: 10.1093/nar/gkw1344 (PMC5416842; doi:10.1093/nar/gkw1344)
Supplement: Supplementary Data [file gkw1344_Supp.pdf]

Wells *et al.*, Figure S1

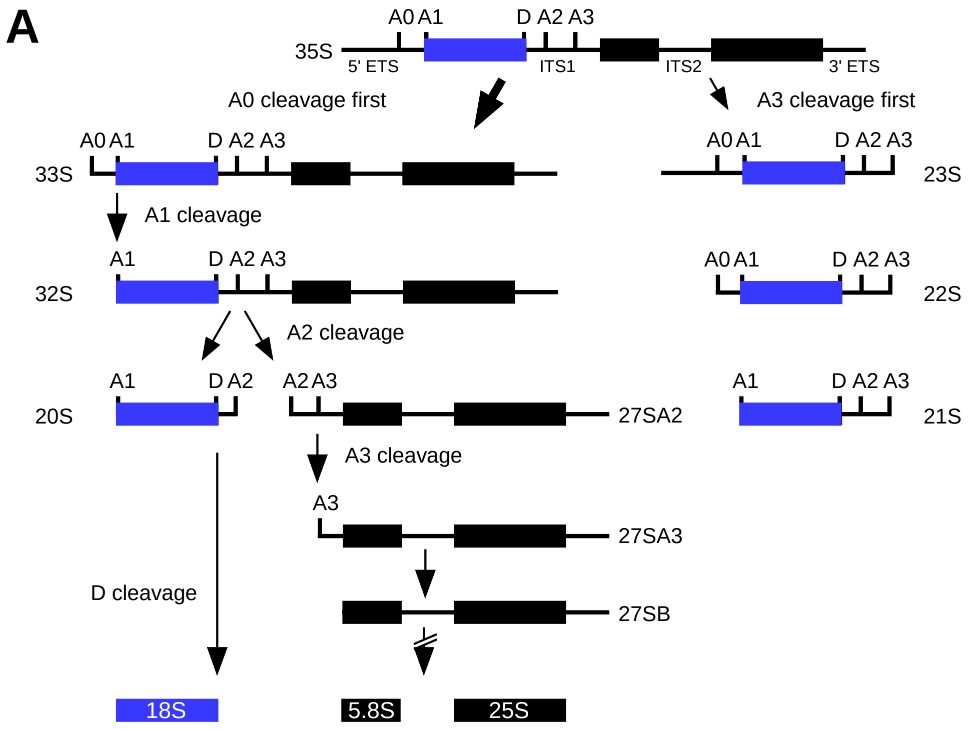

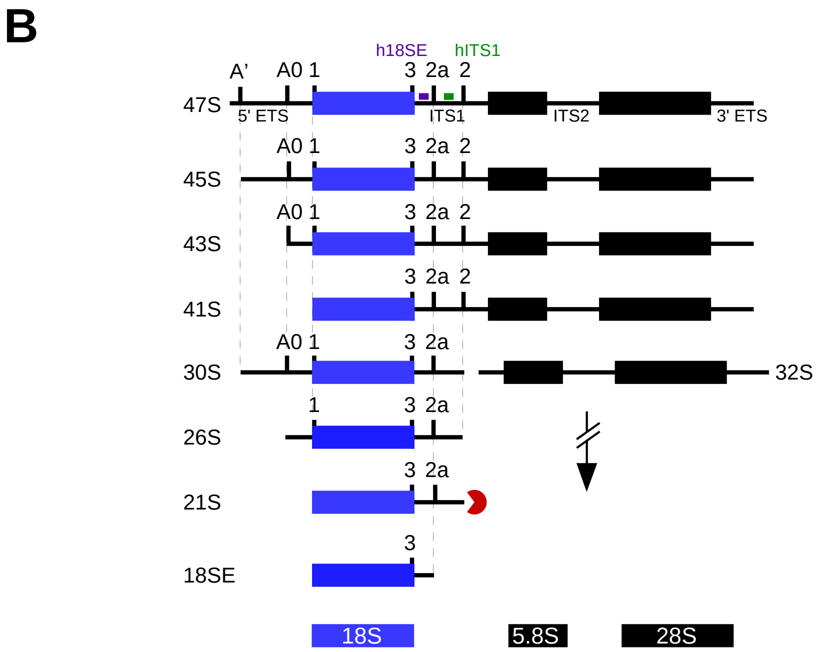

Wells *et al.*, Figure S2

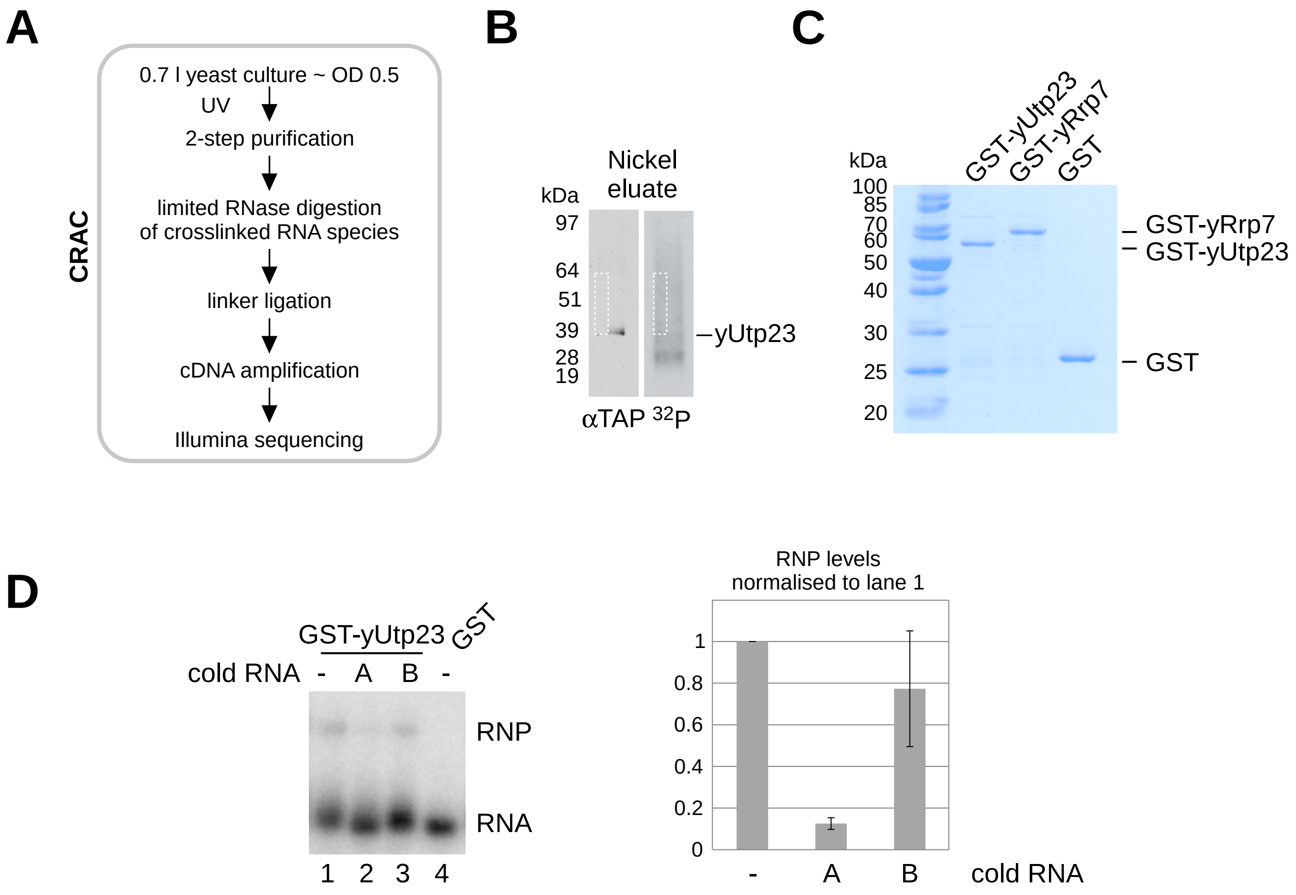

Wells *et al.*, Figure S3

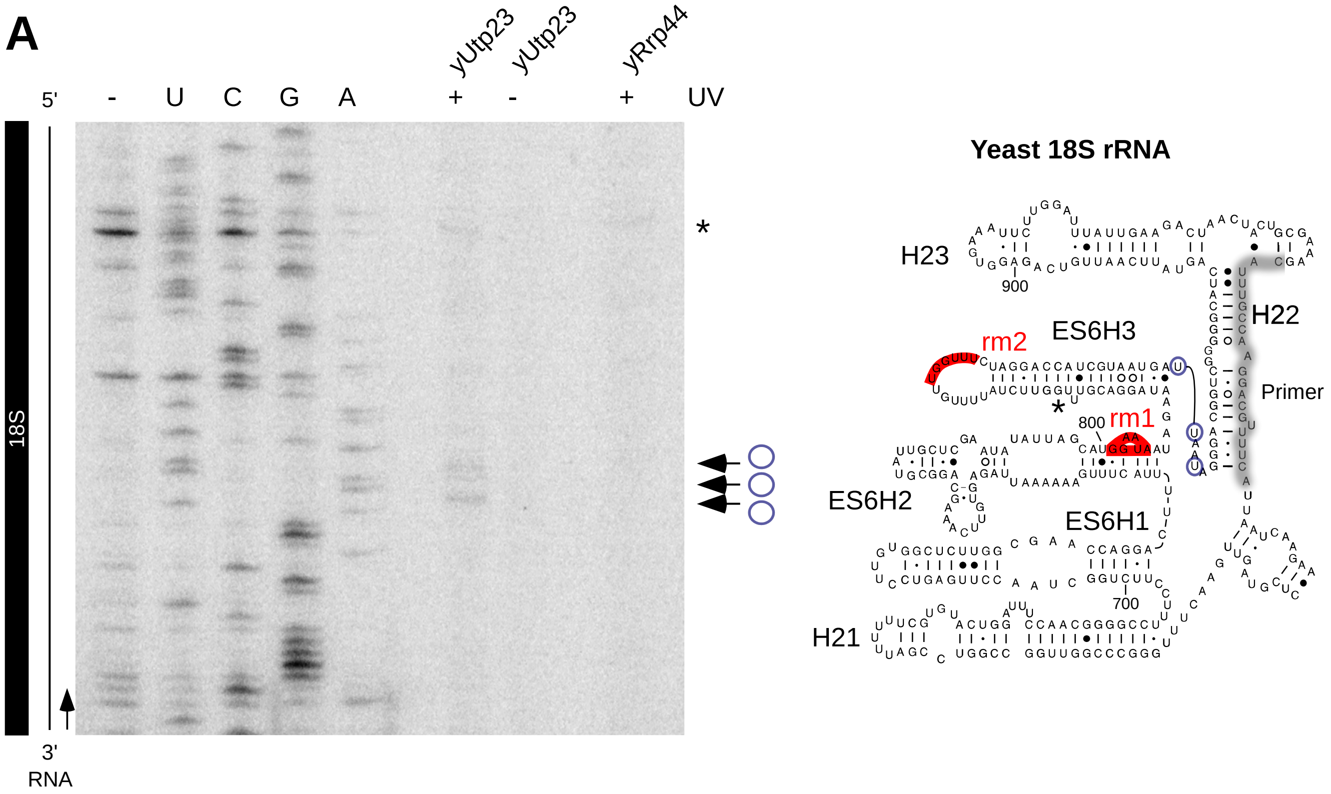

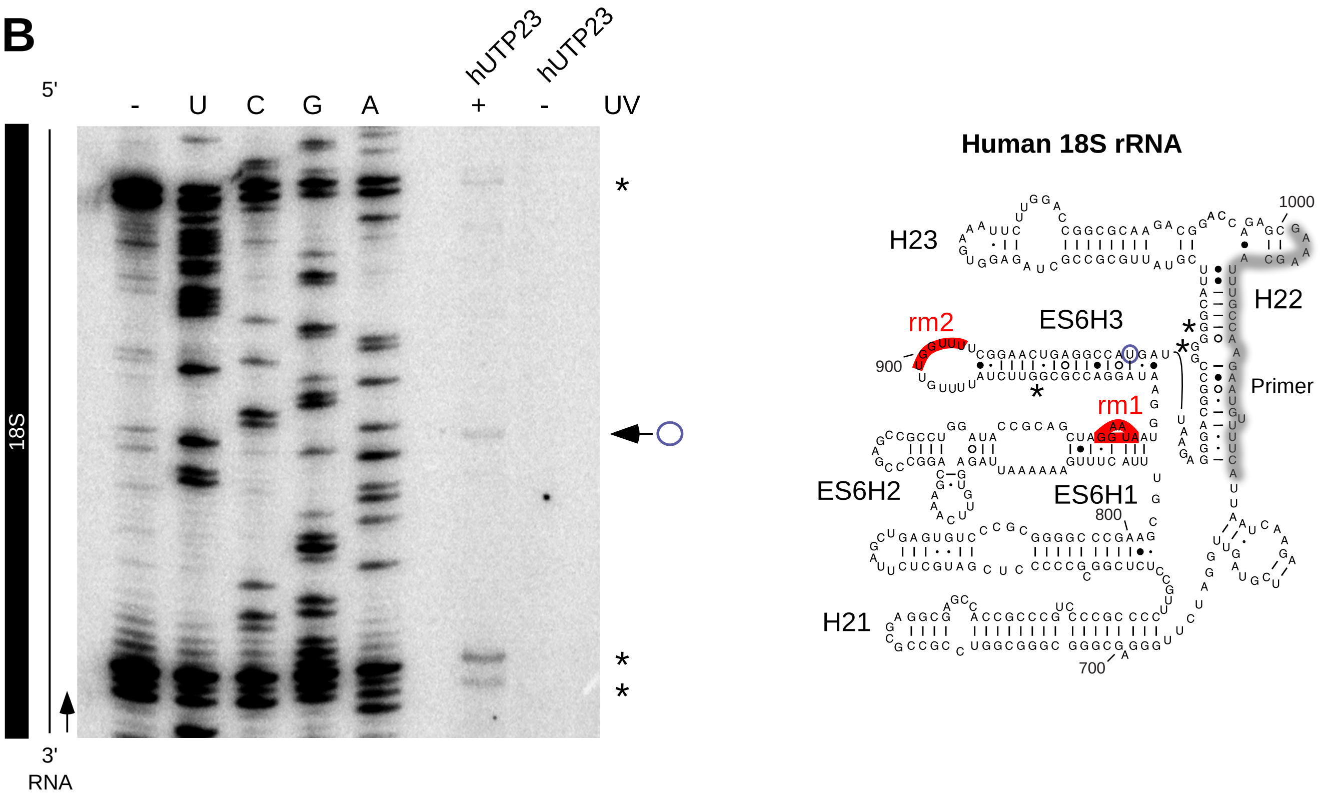

Wells *et al.*, Figure S4

A

Type	Yeast	Human
H/ACA	snR30	U17
CD	snR17a/b/U3	U3
CD	snR128/U14	U14
H/ACA	snR10	NA

B

Top20 snoRNA (%)	Dataset #2	Dataset #4
SNR30	62.9	64.1
SNR17A/B	4.2	2.6
SNR190	3.9	1.5
SNR10	1.9	2.5
SNR128	1.9	0
SNR42	1.7	1.5
SNR86	1.7	1.9
SNR3	1.5	0.8
SNR4	1.2	0
SNR40	1.2	2.3
SNR82	1.1	2.2
SNR33	1	1
SNR31	0.9	0
SNR36	0.9	0
SNR11	0.8	0
SNR35	0.8	0
SNR37	0.8	1.4
SNR41	0.8	0.9
SNR84	0.8	1.5
SNR70	0.7	0.8
SNR13	0	1.7
SNR64	0	1
SNR66	0	1
SNR75	0	2.2
SNR77	0	2
SNR78	0	1.2
others	9.3	5.9

C

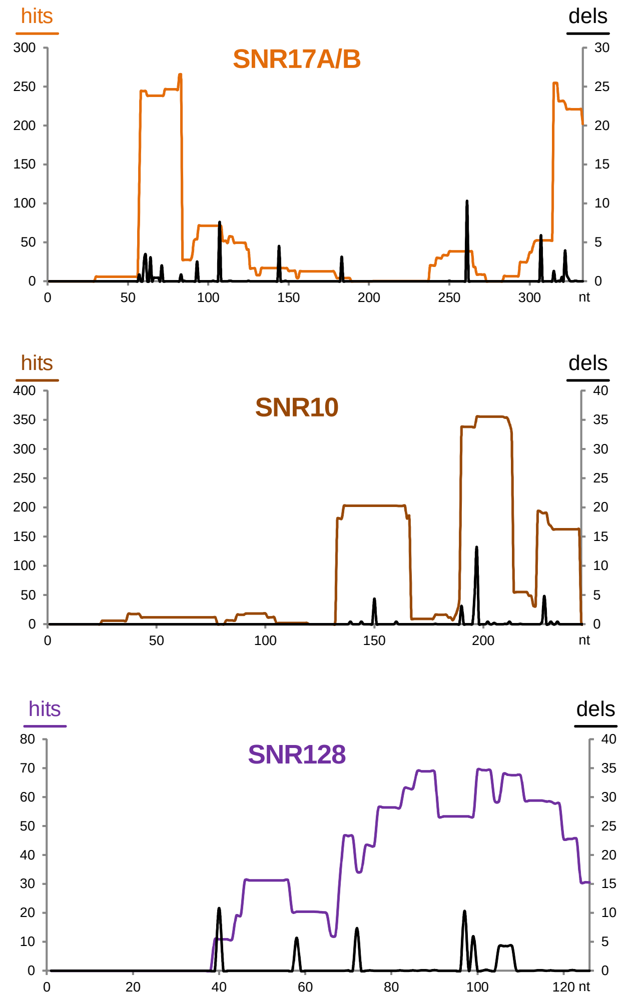

D

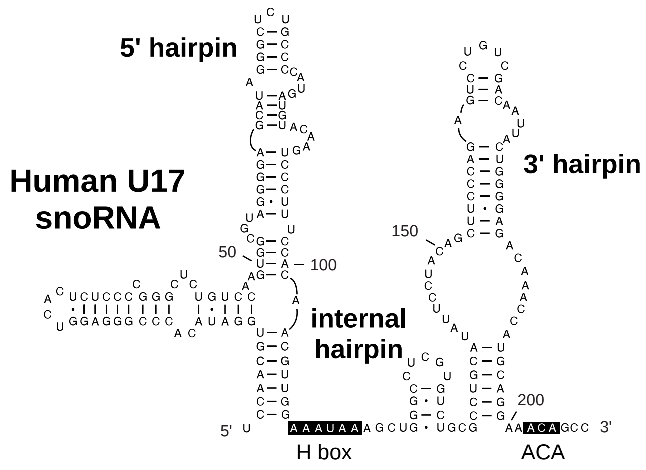

Wells *et al.*, Figure S5

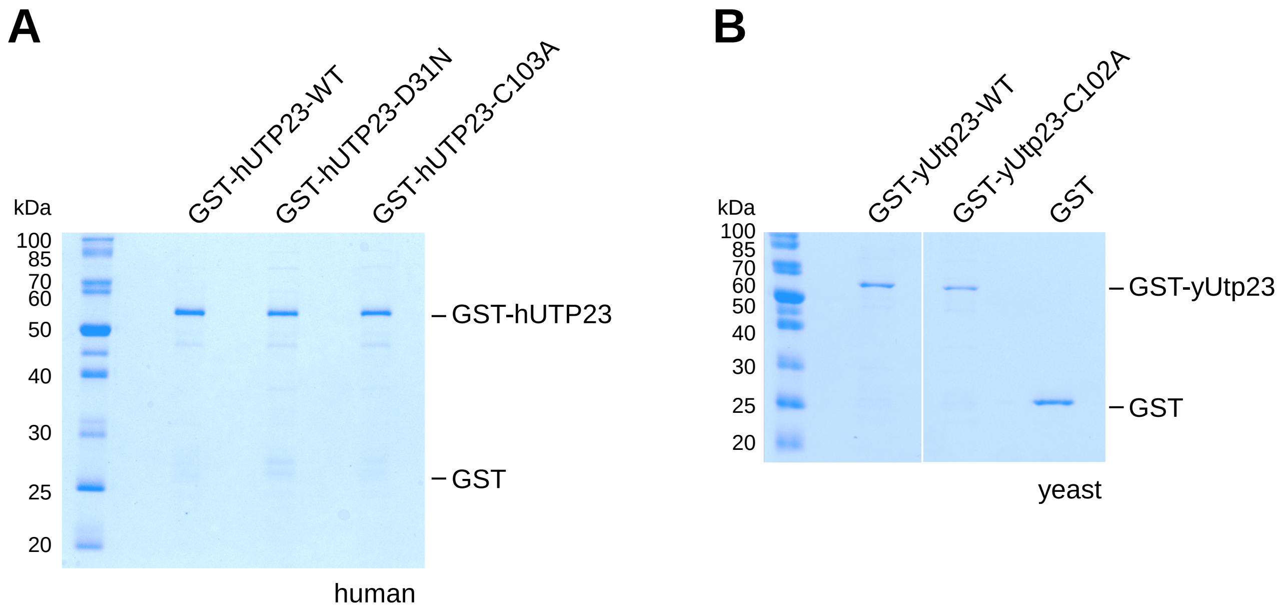

Wells *et al.*, Figure S6

Utp23 CLUSTAL O (1.2.2) multiple sequence alignment

```
                                                  D31N (human hUTP23)
A.thaliana          MRVKRQKKNRRTVRFFTVCYGFRQPYKVLCDGTFVHHLVTNEITPADTAVSELLGGPVKL    60
S.pombe             MRQKRAKNYRKLMHTYQLLFGFREPYQVLVDADFLKDLSQQKIDIQ-AALARTVQGAIKP    59
S.cerevisiae        MRQKRAKSYRKQLLVYSHTFKFREPYQVLVDNQLVLECNNSNFNLP-SGLKRTLQADVKV    59
C.albicans          MRQKRAKAYKKQMSVYVHAFKFREPYQIIVDNELITTCQSASFDIN-KGFTRTIQAENKP    59
D.melanogaster      MKISRFKKSHKTLVFFASNFDYREPYQVLIDATFCQAALQQKIGID-EQIKKYFQCGVKL    59
D.rerio             MKIKRQKHAKKTISFYKYNFSFREPFQILIDGTFCQAALKNKIQIK-EQLPKYLMGEIQL    59
M.musculus          MKITRQKHAKKHLGFFRNNFGVREPYQILLDGTFCQAALRGRIQLR-DQLPRYLMGETQL    59
H.sapiens           MKITRQKHAKKHLGFFRNNFGVREPYQILLDGTFCQAALRGRIQLR-EQLPRYLMGETQL    59
                    *: .* *  :: :  :   :  *:*:::: *  :        :      . . .    :

                                                          C102A (yeast yUtp23)
A.thaliana          FTTRCVIAELEKLGKDFAESLEAAQTLNTATCEHE--EAKTADECLSEVIG--VQNTEHF    116
S.pombe             MITQCCIRQLYSKSDELKQEIRIAKSFERRRCGHI-DEALSPSECIQSVVNINGRNKHRY    118
S.cerevisiae        MITQCCIQALYETRND--GAINLAKQFERRRCNHSFKDPKSPAECIESVVNISGANKHRY    117
C.albicans          MITQCCIQALYDTKNQ--PAIDIAKSFERRKCNHR--EAIDPSQCIESIVNIKGQNKHRY    115
D.melanogaster      LTTQCVILESESLGAPLTGATSIVKRFHVHKCGHE-GKPVPASECIKSMTK--D---NRY    113
D.rerio             CTTNCALKELESLAKDLYGAKLILQRFQIRKCKHM-KDPVPASECLLSMLA--ETNPHHY    116
M.musculus          CTTRCVLKELETLGKELYGAKLIAQKCQVRNCPHF-KSPVSGSECLLSMVD--EGNPHHY    116
H.sapiens           CTTRCVLKELETLGKDLYGAKLIAQKCQVRNCPHF-KNAVSGSECLLSMVE--EGNPHHY    116
                      *.* :                 :  .   * *   .     :*: .:        .::
                                                          C103A (human hUTP23)

A.thaliana          FLGTQDAEFRRKLQQESIVPLVFGLRNILLIDQPSDFQRQSAKDSENKRLTMTDTEKKLL    176
S.pombe             VVATQDPELRQALRSVPGVPLIYMKRSVVILEPASRATLLEKHNKESVQMGMSKEEKLLL    178
S.cerevisiae        VVASQDIDLRRKLRTVPGVPLIHLTRSVMVMEPLSTASAKASKI---------TEEQKLY    168
C.albicans          IVASQDLQLRKKLRKIPGVPLIYMNRSVMVMEPISDVSNQYNMN---------YESKKLT    166
D.melanogaster      VVASQDRLLQESLRKIPGRCLLYLHKATPVLEAPSKASKKWVQRRAKN-L---------M    163
D.rerio             FIATQDQQLTTALKKIPGVPLLYIILNTMVLDKPSERTLKHVEAVQLGEI---------V    167
M.musculus          FVATQDQNLSVKVKRTPGIPLMFIIQNTIVLDKPSPRTVAFVKAVEAGQL---------V    167
H.sapiens           FVATQDQNLSVKVKKKPGVPLMFIIQNTMVLDKPSPKTIAFVKAVESGQL---------V    167
                    .:.:**  :   ::      *:.      :::  *

A.thaliana          VKRTAKIIASNRKEATIANEEWGMPRVVSTKNGLG-VKDRPQFKRNRAKGPNPLSCMKKK    235
S.pombe             SGKKRSANELAID-DQDTKESTDLA-------GTEDSAPKANKKRKGPKGPNPLSIKKRS    230
S.cerevisiae        KGLNDPNIE-KLQ---E---SG--D-------GSGKE-SITKKRKLGPKAPNPLSVKKKK    211
C.albicans          GGLNDIEAG-KLE---KQNEGE--D-------GDGDESEVKKKKRKGPKEPNPLSVKKKK    213
D.melanogaster      LGKQVE----KID---YMKEKQGLK-------PAE-TA-VKPKKHKGPKNPNPLSCKKSK    207
D.rerio             NPAQQK----SLQ---SLKEKEGIS-------GDA-EKRG-RKRKRKQSNPNPLSCLKKK    211
M.musculus          SVHEKQ----SIK---QLKEEQGLV-------RNP-DLRRRRRKKKVGGPNPLSCLKKK    212
H.sapiens           SVHEKE----SIK---HLKEEQGLV-------KNT-EQSR-RKKRKKISGPNPLSCLKKK    211
                                .                              ::     *****  * .

A.thaliana          KENPQSKSKADSNSNAQKEKKEGGSDTQKRSRKRSKKGK-SGPERTE-------------    281
S.pombe             SKNHTTDEPTLPVNIIGD----VGERKKHRRKRK--------------------------    260
S.cerevisiae        KVNSPSDEVKDK----ED----TSKEKKKRRRRKH---KSNTN-------VPVSNGTTAA    253
C.albicans          TDNATAASTNQE----QK----K---KPNRRKRHG---KSKAEEKEDQEQEQVNEATTNE    259
D.melanogaster      KDKAKQQLKGVEQT---------AITKAKRKRVKIPAHVKAALGKD-------------    244
D.rerio             KKKATPQQPKNP-D---------GEKKRKRSRHRK--HKPAGGEQTEVRS----------    249
M.musculus          KKAQDTKSP----A---------SEKKRKRKRIRNRSTLKVSSEQQGAEG----------    249
H.sapiens           KKAPDTQSS----A---------SEKKRKRKRIRNRSNPKVLSEKQNAEGE---------    249
                    .                         . .* : :

A.thaliana          ----------        281
S.pombe             ----------        260
S.cerevisiae        --Q-------        254
C.albicans          DAQEAITATE        269
D.melanogaster      ----------        244
D.rerio             ----------        249
M.musculus          ----------        249
H.sapiens           ----------        249
```

SUPPLEMENTARY FIGURE LEGENDS

Figure S1: Ribosome biogenesis pathways in yeast and humans

Simplified overview of the pre-ribosomal RNA processing pathways in *S. cerevisiae* (**A**) and *H. sapiens* (**B**). Cleavages important for 18S rRNA processing are indicated. The positions of radiolabelled probes used for northern blotting (h18SE and hITS1) are marked above the primary transcript. ETS: external transcribed spacer; ITS: internal transcribed spacer.

Figure S2: *In vivo* RNA-protein crosslinking studies to define yUtp23 binding sites

A Outline of the CRAC crosslinking technique (UV crosslinking and analysis of cDNAs).

B Proteins purified from an UV-crosslinked yeast strain expressing C-terminally HTP-tagged yUtp23 were separated by SDS-PAGE and visualised by immunoblotting using the anti-TAP antibody (left panel), which recognises the C-terminus of the HTP construct after TEV cleavage. Crosslinked RNA fragments, which were co-purified with the yUtp23 protein, were radioactively labelled and detected by autoradiography (right panel). Box: membrane cut for isolation of crosslinked RNA.

C Recombinant, GST-tagged yUtp23, yRrp7 or free GST used for electromobility shift assays (EMSA) or protein-protein interaction studies were expressed in *E. coli* and purified using glutathione sepharose. Proteins were separated by SDS-PAGE and stained with Coomassie blue.

D EMSA showing the binding of 5000 nM GST-yUtp23 or GST to trace amounts of an *in vitro* transcribed radiolabelled 18S rRNA ES6 fragment (nt 775-963, RNA A). Binding was performed in the absence (lanes 1 and 4) or with an excess (3 pmoles) of non-radiolabelled competitor RNA A (18S nt 775-963, lane 2) or RNA B (18S nt 1022-1146, lane 3). RNP complexes were separated from unbound RNA on 4% native polyacrylamide gels and visualised using a PhoshorImager. RNP levels from three independent experiments were determined by quantification using ImageQuant and normalised to the reaction containing no competitor RNA (lane 1).

Figure S3: Primer extension analysis of yUtp23/hUTP23 crosslinking sites in the yeast and human 18S ES6 region

A RNA-containing complexes were isolated from yeast strains expressing C-terminally HTP-tagged yUtp23, or the exosome component yRrp44 as a control. Yeast cells were either crosslinked (100 sec at 254 nm) (+UV) or non-treated (-UV), and purifications were performed as described for CRAC, with the exception that purified complexes were not treated with RNase. Briefly, complexes were first purified on IgG sepharose, eluted by TEV cleavage and re-purified on Nickel agarose under denaturing conditions (1,2). Co-purified RNA was extracted and analysed by primer extension using a primer hybridising to nt 943-

963 of the mature yeast 18S rRNA (left panel). *In vitro* transcribed RNA mimicking the yeast 23S rRNA was used to generate a sequencing ladder. Reverse transcriptase stops that are only present on RNA isolated from crosslinked yUtp23 complexes are marked by circles, a stop also present in the crosslinked control sample (yRrp44) is marked by an asterisk. Right panel: Predicted structure of the yeast 18S ES6 region after snR30 release, highlighting the positions of the primer (shaded in grey) and the reverse transcriptase stops shown in the left panel (circles and asterisk).

B RNA-containing complexes were isolated from crosslinked (+UV) or non-treated (-UV) HEK293 cells expressing C-terminally His_8-PP-2xHA-tagged wild type hUTP23. UV crosslinking was performed in the Stratalinker (3 x 800 mJ/cm^2) and purifications were performed from sonicated whole-cell extracts as described for CRAC (1,2), with the exception that purified complexes were not treated with RNase. Briefly, complexes were first purified on anti-HA agarose, eluted by PreScission protease cleavage and then re-purified on Nickel agarose under denaturing conditions. Co-purified RNA was extracted and analysed by primer extension using a primer hybridising to nt 1001-1026 of the mature human 18S rRNA (left panel). Total RNA extracted from HEK293 cells was used to generate a sequencing ladder. Reverse transcriptase stops visible in all lanes except the non-crosslinked hUTP23 sample are likely due to secondary structure of the 18S rRNA and marked by asterisks. Circle: Reverse transcriptase stop that is only present in RNA isolated from crosslinked hUTP23 complexes. Right panel: Predicted structure of the human 18S ES6 region after U17 release, highlighting the positions of the primer (shaded in grey) and the reverse transcriptase stops shown in the left panel (circle and asterisks).

Figure S4: yUtp23 crosslinking sites on small nucleolar RNAs (snoRNAs)

A Yeast and human snoRNAs with non-modifying roles important for A0, A1/1 and A2/2a cleavages

B Top 20 snoRNA hits. Sequence data from two individual yUtp23 datasets are shown as percentages of all reads mapped to snoRNAs.

C yUtp23 crosslinking sites on three snoRNAs involved in SSU production are plotted as total reads (hits) or microdeletions (dels) per million mapped sequences. Top: SNR17A/B (U3); middle: SNR10 and bottom: SNR128 (U14). Data from the two individual datasets shown in panel **B** were combined.

D Predicted secondary structure of the human U17 box H/ACA snoRNA. The H and ACA boxes are shown in black.

Figure S5: Recombinant proteins used for protein-protein interaction and RNA binding assays

Recombinant, GST-tagged wild type or mutant Utp23 proteins from human (hUTP23, **A**) or yeast (yUtp23, **B**) or the GST tag alone (**B**) were expressed in *E. coli* and purified using glutathione sepharose. Proteins were separated by SDS-PAGE and stained with Coomassie blue. D31N: hUTP23 PIN mutant. C103A: hUTP23 Zinc finger mutant. C102A: yUtp23 Zinc finger mutant.

Figure S6: Utp23 protein alignment

Selected plant, fungal and metazoan protein sequences retrieved by a NCBI protein BLAST search using the *S. cerevisiae* yUtp23 (YOR004W) protein sequence were aligned with clustalO (http://www.ebi.ac.uk/Tools/msa/clustalo/). The alignment was modified to highlight acidic residues (red) within the PIN domain predicted catalytic centre (shaded in yellow) and conserved residues in the CCHC Zinc finger motif (blue), respectively.

SUPPLEMENTARY TABLES

Table S1: Yeast strains used in this study

BY4741	*MATa; his3Δ1; leu2Δ0; met15Δ0; ura3Δ0*	(Euroscarf)
yUtp23-HTP	*MATa; his3Δ1; leu2Δ0; met15Δ0; ura3Δ0; UTP23-HTP-URA3*	(this study)

Table S2: Plasmids used in this study

Insert	Vector	Purpose	
Yeast			
GST-yUtp23-WT	pGEX-6P1	To purify recombinant protein	(this study)
GST-yUtp23-C102A	pGEX-6P1	To purify recombinant protein	(this study)
GST-yUtp24	pGEX-6P1	To purify recombinant protein	(3)
GST-yRrp7	pGEX-6P1	To purify recombinant protein	(this study)
ProtA-TEV-yUtp23-His_6	Nzztev80 (4)	To purify recombinant protein	(this study)
His_6-yUtp23	pET100	To produce *in vitro* translates	(this study)
His_6-yRok1	pET100	To produce *in vitro* translates	(this study)
His_6-yDhr1	pET100	To produce *in vitro* translates	(this study)
His_6-yKri1	pET100	To produce *in vitro* translates	(this study)
His_6-yNhp2	pET100	To produce *in vitro* translates	(this study)
His_6-yRrp9	pET100	To produce *in vitro* translates	(this study)
pT7snR30 (cut BamHI)	pSP6T7	For *in vitro* transcription (T7 Pol)	(5)
Human			
hUTP23-WT-His_8-PP-2HA	pcDNA5	To stably transfect HEK293 cells	(this study)
hUTP23-D31N-His_8-PP-2HA	pcDNA5	To stably transfect HEK293 cells	(this study)
hUTP23-C103A-His_8-PP-2HA	pcDNA5	To stably transfect HEK293 cells	(this study)
GST-hUTP23-WT	pGEX-6P1	To purify recombinant protein	(this study)
GST-hUTP23-D31N	pGEX-6P1	To purify recombinant protein	(this study)
GST-hUTP23-C103A	pGEX-6P1	To purify recombinant protein	(this study)
His_6-hUTP24	pET100	To purify recombinant protein	(this study)
His_6-hRRP7	pET100	To purify recombinant protein	(this study)
His_6-hNHP2	pET100	To purify recombinant protein	(this study)
His_6-hROK1/DDX52	pET100	To produce *in vitro* translates	(this study)
pBS+HU17	pBSIISK+	To amplify U17 for transcription with T7 Pol (see Table S3)	(6)

Table S3: Oligonucleotides (5'-3') used in this study

**Strain construction**	
**yUtp23-HTP**	
F	GCAACACTAACGTCCCCGTTTCAAATGGGACCACAGCCGCGCAG GAGCACCATCACCATCACC
R	CGCAATTATATTCAAAAGATTGGCCAGTCAATTAACTTAAGGAAAA AATTACGACTCACTATAGGG
**CRAC**	
3'-Linker	5'- rAppTGGAATTCTCGGGTGCCAAGG/ddC/ -3' (miRCat-33™)
5'-Linker (L5Aa)	5'-invddT-ACACrGrArCrGrCrUrCrUrUrCrCrGrArUrCrU rNrNrNrTrArArGrC-OH-3'
5'-Linker (L5Ac)	5'-invddT-ACACrGrArCrGrCrUrCrUrUrCrCrGrArUrCrU rNrNrNrGrCrGrCrArGrC-OH-3'
5'-Linker (L5f)	5'-invddT-ACACrGrArCrGrCrUrCrUrUrCrCrGrArUrCrU rGrCrGrArGrC-OH-3'
RT	CCTTGGCACCCGAGAATT (miRCat-33™)
PCR - F	AATGATACGGCGACCACCGAGATCTACACTCTTTCCCTACACGAC GCTCTTCCGATCT
PCR - R	CAAGCAGAAGACGGCATACGAGATCGGTCTCGGCATTCCTGGCC TTGGCACCCGAGAATTCC
**Cloning**	**(Vector)**
Yeast	
**yUtp23**	**(pET100 and pGEX-6P1)**
F (BamHI)	CACCGGATCCATGCGTCAAAAGAGGGCTAAGTCATATAGG
R (SalI)	GTCGACTTACTGCGCGGCTGTGGTC
**yUtp23**	**(Nzztev80) (4)**
F (Sph1)	TTCAGGGCATGCTGGAAGTTCTGTTCCAGGGACCCCGTCAAAAG AGGGCTAAGTCATATAG
R (XmaI)	CGTCGACCCGGGGCTGCGCGGCTGTGGTC
**yRrp7**	**(pGEX-6P1)**
F (BamHI)	GCGCGGATCCATGGGTATTGAAGACATTAGCGC
R (SalI)	CCCCGTCGACTTAAGTGTATGGATTGAATTTTCTCTTAGC
**yRok1**	**(pET100)**
F (BglII)	CACCAGATCTATGGATATTTTTAGAGTATTAACTAGAGGAGC
R (XhoI)	CTCGAGTTATTTCGAGAAATGTTTTTTTGAAAG
**yDhr1**	**(pET100)**
F (BglII)	CACCAGATCTATGGGTACTTACAGAAAAAGGTTTAATG
R (XhoI)	CTCGAGTTATTTTTTTTCTTTCTCTTCACCTGTG
**yKri1**	**(pET100)**
F (BglII)	CACCAGATCTATGCCAAGAAAAAAGTCTGCC
R (XhoI)	CTCGAGTTACTTTTTCTGGTGGCCTTTATG
**yNhp2**	**(pET100)**
F (BamHI)	CACCGGATCCATGGGTAAAGACAACAAGGAACATAAG
R (SalI)	GTCGACTCATAAAGCTTGAACTTCTTTGACAAC

**yRrp9**	**(pET100)**
F (BamHI)	CACCGGATCCATGTCAGATGTTACCCAACAGAAAAAGAG
R (SalI)	GTCGACCTAAAAGCCTGTTTGGTCAATGACAG
Human	
**hUTP23**	**(pGEX-6P1)**
F (BamHI)	CACCGGATCCATGAAGATCACAAGGCAGAAACATGCC
R (XhoI)	CGCGCTCGAGTCATTCTCCTTCTGCATTCTGCTTCTC
**hUTP24**	**(pET100)**
F (BglII)	CACCAGATCTATGGGGAAGCAAAAGAAAACAAGGAAGTATGCGA CCATGAAGCGAATGCTTAG
R (XhoI)	CGCGCTCGAGTTAGAATCGAGGGGCTCCATAATCATCTGG
**hRRP7**	**(pET100)**
F (BamHI)	CACCGGATCCATGGTGGCGCGCAGGAGGAAGTGCGC
R (XhoI)	CGCGCTCGAGTCAGTACGGTCGGAATTTGCGC
**hNHP2**	**(pET100)**
F (BamHI)	CACCGGATCCATGACCAAAATAAAGGCAGATCC
R (XhoI)	CTCGAGTTAAAGGGGTAGGGGCAGG
**hROK1/DDX52**	**(pET100)**
F (BamHI)	CACCGGATCCATGGACGTCCACGATCTCTTTC
R (XhoI)	CTCGAGTTAACTTTTGTCTTCAAGAGCTACTTTC
**Mutagenesis**	**(Mutation)**
Yeast	
**yUtp23**	**C102A (Zinc finger mutant)**
F	CAAAGTCACCTGCGGAGGCCATCGAAAGCGTCGTTAA
R	TTAACGACGCTTTCGATGGCCTCCGCAGGTGACTTTG
**snR30 ΔIH**	**To delete the IH of snR30 (nt 398-531)**
F	GTTTAACTTAGATTAAGCCGCAGTATATTCCTAAACACTATGAAAT GAC
R	GGAATATACTGCGGCTTAATCTAAGTTAAACTCGTCAACGGGG
Human	
**hUTP23**	**D31N (PIN mutant)**
F	CCGTACCAGATCCTGCTGAACGGCACCTTCTGTCAG
R	CTGACAGAAGGTGCCGTTCAGCAGGATCTGGTACGG
**hUTP23**	**C103A (Zinc finger mutant)**
F	GCAGTGAGTGGATCAGAAGCTCTGCTTTCCATGGTTGAAGAG
R	CTCTTCAACCATGGAAAGCAGAGCTTCTGATCCACTCACTGC
**Northern blotting**	
hITS1 (6121)	AGGGGTCTTTAAACCTCCGCGCCGGAACGCGCTAGGTAC
h18SE	CCTCGCCCTCCGGGCTCCGTTAATGATC
U17	TTCCTGCATGGTTTGTCTCC
7SK	GTGTCTGGAGTCTTGGAAGC
**Primer extension**	
y18S A963R	TGAAAACGTCCTTGGCAAATG
h18S A1026R	TGAAAACATTCTTGGCAAATGCTTTC

For transcription	
	**(yeast 18S rRNA: nt 775-963)**
F	CGGAATTCTAATACGACTCACTATAGGCGTATTGCTCGAATATATTAGC (T7 promoter)
R	TGAAAACGTCCTTGGCAAATG
	**(yeast 18S rRNA: nt 1022-1146)**
F	CGGAATTCTAATACGACTCACTATAGGCATAAACTATGCCGACTAGGGATC (T7 promoter)
R	CAATTCCTTTAAGTTTCAGCCTTG
	**(yeast 23S rRNA: nt 1-2786)**
F	CGGAATTCTAATACGACTCACTATAGGATGCGAAAGCAGTTGAAGAC (T7 promoter)
R	GTTTGTTACCTCTGGGCCCC
	**(snR30 fragments)**
F (nt 1)	CGGAATTCTAATACGACTCACTATAGGAACCATAGTCTCGTGCTAGTTCGG (T7 promoter)
F (nt 398)	CGGAATTCTAATACGACTCACTATAGGGTAGGACGCATGATCTTGAGCTC (T7 promoter)
F (nt 531)	CGGAATTCTAATACGACTCACTATAGGCCGCAGTATATTCCTAAACACTATG (T7 promoter)
R (nt 398)	CTTAATCTAAGTTAAACTCGTCAACGG
R (nt 531)	GTAGGACGAACAACAAAGATGACC
R (nt 606)	AGATGTCTGCAGTATGGTTTTACCC
	**(U17 snoRNA)**
F	GCGTAATACGACTCACTATAGGGCGATCCAACGTGGATACACCCGG (T7 promoter)
R	GGCTGTTTCCTGCATCGGTTTGTC

Table S4: siRNAs used in this study

Gene	Target Sequence (5'-3')	Source
Luciferase GL2	CGUACGCGGAAUACUUCGA	Eurofins MWG (7)
hUTP23	GAAAGUAUCAAACAUCUCAUU	Eurofins MWG

Table S5: Antibodies used in this study

Target	Source	Cat. No.	Dilution	Species
**Western blotting**				
α-hKaryopherin	Santa Cruz	sc-11367	1:1000	rabbit
α-hUTP23	Aviva	ARP60526_P050	1:1000	rabbit
α-HA	Babco	MMS-101P	1:5000	mouse
α-TAP*	Thermo Scientific	CAB1001	1:10,000	rabbit
α-mouse-HRP	Santa Cruz	sc-2316	1:10,000	donkey
α-rabbit-HRP	Santa Cruz	sc-2313	1:10,000	donkey
α-rabbit-IRdye 800CW	Li-Cor	926-32211	1:10,000	goat
**Immunofluorescence**				
α-HA	Babco	MMS-101P	1:500	mouse
α-hFibrillarin	Santa Cruz	sc-25397	1:200	rabbit
α-rabbit-IgG (Alexa Fluor 555 conjugate)	Invitrogen	A-31572	1:500	donkey
α-mouse-IgG (Alexa Fluor 555 conjugate)	Invitrogen	A-31570	1:500	donkey

***** The α-TAP antibody recognises the C-terminus of the HTP construct before and after TEV cleavage.

SUPPLEMENTARY REFERENCES

1. Granneman, S., Kudla, G., Petfalski, E. and Tollervey, D. (2009) Identification of protein binding sites on U3 snoRNA and pre-rRNA by UV cross-linking and high-throughput analysis of cDNAs. *Proc Natl Acad Sci U S A*, **106**, 9613-9618.
2. Granneman, S., Petfalski, E. and Tollervey, D. (2011) A cluster of ribosome synthesis factors regulate pre-rRNA folding and 5.8S rRNA maturation by the Rat1 exonuclease. *EMBO J*, **30**, 4006-4019.
3. Wells, G.R., Weichmann, F., Colvin, D., Sloan, K.E., Kudla, G., Tollervey, D., Watkins, N.J. and Schneider, C. (2016) The PIN domain endonuclease Utp24 cleaves pre-ribosomal RNA at two coupled sites in yeast and humans. *Nucleic Acids Res*, **44**, 5399-5409.
4. Mingot, J.M., Bohnsack, M.T., Jakle, U. and Gorlich, D. (2004) Exportin 7 defines a novel general nuclear export pathway. *EMBO J*, **23**, 3227-3236.
5. Watkins, N.J., Gottschalk, A., Neubauer, G., Kastner, B., Fabrizio, P., Mann, M. and Luhrmann, R. (1998) Cbf5p, a potential pseudouridine synthase, and Nhp2p, a putative RNA-binding protein, are present together with Gar1p in all H BOX/ACA-motif snoRNPs and constitute a common bipartite structure. *RNA*, **4**, 1549-1568.
6. Watkins, N.J., Leverette, R.D., Xia, L., Andrews, M.T. and Maxwell, E.S. (1996) Elements essential for processing intronic U14 snoRNA are located at the termini of the mature snoRNA sequence and include conserved nucleotide boxes C and D. *RNA*, **2**, 118-133.
7. Elbashir, S.M., Harborth, J., Weber, K. and Tuschl, T. (2002) Analysis of gene function in somatic mammalian cells using small interfering RNAs. *Methods*, **26**, 199-213.
